# Penaeidins restrict white spot syndrome virus infection by antagonizing the envelope proteins to block viral entry

**DOI:** 10.1080/22221751.2020.1729068

**Published:** 2020-02-20

**Authors:** Bang Xiao, Qihui Fu, Shengwen Niu, Peng Zhu, Jianguo He, Chaozheng Li

**Affiliations:** a Southern Marine Science and Engineering Guangdong Laboratory (Zhuhai)/ School of Marine Sciences, Sun Yat-sen University, Guangzhou, P. R. People’s Republic of China; b State Key Laboratory of Biocontrol/ School of Life Sciences, Sun Yat-sen University, Guangzhou, P. R. People’s Republic of China; c Guangxi Key Laboratory of Beibu Gulf Marine Biodiversity Conservation, Beibu Gluf University, Qinzhou, P. R. People’s Republic of China

**Keywords:** Penaeidin, shrimp, WSSV, antiviral mechanism, viral internalization

## Abstract

Emerging studies have indicated that some penaeidins restrict virus infection; however, the mechanism(s) involved are poorly understood. In the present study, we uncovered that penaeidins are a novel family of antiviral effectors against white spot syndrome virus (WSSV), which antagonize the envelope proteins to block viral entry. We found that the expression levels of four identified penaeidins from *Litopenaeus vannamei*, including *BigPEN*, *PEN2*, *PEN3*, and *PEN4*, were significantly induced in hemocytes during the early stage of WSSV infection. Knockdown of each penaeidin *in vivo* via RNA interference resulted in elevated viral loads and rendered shrimp more susceptible to WSSV, while the survival rate was rescued via the injection of recombinant penaeidins. All penaeidins, except PEN4, were shown to interact with several envelope proteins of WSSV, and all four penaeidins were observed to be located on the outer surface of the WSSV virion. Co-incubation of each recombinant penaeidin with WSSV inhibited virion internalization into hemocytes. More importantly, we found that PEN2 competitively bound to the envelope protein VP24 to release it from polymeric immunoglobulin receptor (pIgR), the cellular receptor required for WSSV infection. Moreover, we also demonstrated that BigPEN was able to bind to VP28 of WSSV, which disrupted the interaction between VP28 and Rab7 – the Rab GTPase that contributes to viral entry by binding with VP28. Taken together, our results demonstrated that penaeidins interact with the envelope proteins of WSSV to block multiple viral infection processes, thereby protecting the host against WSSV.

## Introduction

The host innate immune system plays a significant role in protecting the organism from pathogenic invasion, particularly in invertebrates, which lack adaptive immunity. Antimicrobial peptides (AMPs) establish a first line of defense against a large spectrum of pathogens, such as bacteria, fungi, parasites, and viruses [1]. In general, the antimicrobial mechanism of action of AMPs is thought to involve direct membrane disruption [2,3]. However, their mechanism of action in defending against viral agents is not very well established. There is growing evidence that the direct interactions between AMPs and structural components of the virion could be a common inhibitory mechanism for destroying or destabilizing the virus and rendering it non-infectious [3]. In particular, AMPs exhibit antiviral activity by targeting any stage within the life cycle of the virus, including attachment to receptors on the cell surface, endocytic uptake and trafficking, uncoating of the genome, viral replication, and particle assembly and release. For example, human cathelicidins prevent the influenza virus from infecting host cells by directly binding the virus and destroying its membrane [4]. The mammalian defensin HNP1 directly binds HIV-1 and alters HIV-1 fusion through interactions with glycoprotein-41 [5]. Additionally, human alpha defensins have been shown to bind and stabilize the virus capsid and block adenovirus uncoating to neutralize infection [6]. Human defensins HNP2 and HD5 have been shown to bind HSV-2 DNA, indicating that these defensins may be able to inhibit infection by blocking gene expression via a post-transcriptional block [7]. In addition, HNP1–3 was shown to inhibit reverse transcription and integration by inhibiting PKC activity, which is important for assembly of the virions [8].

White spot syndrome virus (WSSV) is an enveloped virus with a dsDNA genome of approximately 300 kbp [9]. WSSV has a remarkably wide host range, with up to 98 species identified, and has caused enormous economic losses, especially in the shrimp aquaculture [10]. A model of the WSSV life cycle and morphogenesis primarily includes three phases: (1) entry into the host cell, (2) uncoating of the genome followed by replication, and (3) particle assembly and release [9]. The first stage of viral entry into the host cell has been thoroughly studied and involves a wide range of molecular interactions between the WSSV envelope proteins and the host [11]. For example, the major envelope protein VP28 of WSSV plays a vital role in viral entry into the host cells by interacting with several host factors, such as the C-type lectin isolated from the stomach of *Marsupenaeus japonicus* (MjsvCL) shrimp [12] and the Rab7 GTPase from *Penaeus monodon* (PmRab7) shrimp [13]. Recently, an exciting study identified the poly-immunoglobin receptor from *M. japonicas* (MjpIgR) as the transmembrane receptor for WSSV infection, which interacts with the envelope protein VP24 and facilitates viral entry [14]. In addition, other envelope proteins, including VP53A, VP31, and VP187, have also been found to interact with different host factors to facilitate viral infection [15–17]. All stages of viral infection are suitable targets for the development of antiviral agents to prevent WSSV infection in farmed shrimp [18]. AMPs are one class of such agents that significantly block multiple stages within the life cycle of many human and animal viruses [1]. Thus, acquiring a more thorough understanding of antiviral agents, such as AMP-mediated defense mechanisms in shrimp, may aid in developing new strategies and methods for the treatment and prevention of WSSV infection.

Penaeidins, which belong to an AMP family initially characterized in the shrimp *Litopenaeus vannamei*, play a significant role in antibacterial immunity [19]. Penaeidins are unique cationic molecules that consist of an N-terminal proline-rich region (PRR) and a C-terminal cysteine-rich region (CRR) with six conserved cysteine residues that form three disulfide bonds [19]. Based on amino acid sequence comparisons and the position of specific amino acids, penaeidins have been previously classified into four distinct subgroups: PEN2, PEN3, PEN4, and PEN5 (as PEN1 turned out to be the variant of PEN2) [20]. The mRNA level of *PmPEN5* from *P. monodon* was significantly induced upon viral infection, suggesting its possible role in shrimp antiviral immunity [21]. However, the antiviral mechanisms of AMPs from invertebrates, including penaeidins, remain elusive.

In this study, we examined the mechanism of inhibition of WSSV infection by penaeidins. We found that penaeidins have potent anti-WSSV activity. These molecules block WSSV infection by disrupting the interactions of viral envelope proteins with essential host factors, such as VP28–Rab7 and VP24-pIgR, thereby preventing virus-mediated entry into host cells. These studies reveal the antiviral mechanism of penaeidins, which highlight a new antiviral strategy that has the potential to be employed to control WSSV infection.

## Materials and methods

### Purification of intact WSSV viral particles

Virions were isolated from WSSV-infected shrimp following a previously published method [22]. The tissues of infected *L. vannamei* shrimp, excluding the hepatopancreas, were collected on ice. Ten grams of infected tissues were homogenized in 500 ml of TNE buffer (50 mM Tris-HCl, 400 mM NaCl, 5 mM EDTA, pH 8.5) containing a combination of protease inhibitors (1 mM phenylmethylsulfonyl fluoride (PMSF), 1 mM benzamidine, and 1 mM Na_2_S_2_O_5_) and then centrifuged at 5000×*g* for 5 minutes at 4°C. After filtering with a nylon net (400 mesh), the supernatant was centrifuged at 30,000×*g* for 30 minutes at 4°C. Then the upper loose pellet was rinsed out carefully, and the lower white pellet was suspended in 10 ml TM buffer (50 mM Tris–HCl, 10 mM MgCl_2_, pH 7.5). After centrifugation at 5000×*g* for 5 minutes, the virus particles were sedimented by centrifugation at 30,000×*g* for 20 minutes at 4°C and then resuspended and stored in 1 ml TM buffer containing 0.1% NaN_3_. The degree of isolated virus purity was evaluated by negative-staining transmission electron microscopy (TEM) (JEM-100CXII, JEOL, Tokyo, Japan).

### Animals and pathogens

Healthy *L. vannamei* (approximately 4–6 g each) were purchased from a local shrimp farm in Zhanjiang, Guangdong Province, China, and cultured in a recirculating water tank system filled with air-pumped sea water with 2.5% salinity at 27°C. The shrimp were fed to satiation three times per day with a commercial diet (Haid Group, Guangzhou, China). Before all experimental treatments, shrimp (5% of total) were analyzed and confirmed to be free of white spot syndrome virus (WSSV) and *V. parahaemolyticus* by PCR or RT–PCR methods according to published standard operating procedures [23]. The Gram-negative *V. parahaemolyticus* (ATCC 17802, purchased from Guangdong Culture Collection Center, China) were cultured in Luria broth (LB) medium (BD Biosciences, San Jose, California, USA) overnight at 37°C. Bacteria were quantified by counting the colony-forming units (CFU) per milliliter on LB agar plates. The final injection concentration of *V. parahaemolyticus* was approximately 1 × 10^5^ CFU/50 μl. The WSSV (Chinese strain, AF332093) was extracted from the WSSV-infected shrimp muscle tissue and stored at −80°C. Before injection, muscle tissue was homogenized and prepared as a WSSV inoculum with approximately 1 × 10^5^ copies in 50 μl PBS (140 mM NaCl, 2.7 mM KCl, 10 mM Na_2_HPO_4_, 1.8 mM KH_2_PO_4_, pH 7.4). In the pathogenic challenge experiments, each shrimp received an intraperitoneal injection of 50 µl WSSV or *V. parahaemolyticus* solution in the second abdominal segment using a 1-ml syringe.

### RNA and genomic DNA extraction and cDNA synthesis

Total RNA was extracted from different tissues of shrimp using the RNeasy Mini kit (Qiagen, Hilden, Germany). The genomic DNA was extracted from shrimp tissues using a TIANGEN Marine Animals DNA Kit (TIANGEN, Guangzhou, China), according to the manufacturer's instructions. First-strand cDNA synthesis was performed using a cDNA Synthesis Kit (Takara, Dalian, China), following the manufacturer's instructions.

### Cloning of shrimp penaeidins

The partial cDNA sequence of *BigPEN* was obtained from transcriptomic sequencing of *L. vannamei* [24], and its full-length cDNA sequence was cloned by 5′ and 3′ rapid amplification of cDNA ends (RACE) PCR according to a previously published method [25]. The full-length sequence of *PEN2* (accession no. DQ206401), *PEN3* (accession no. DQ206403), and *PEN4* (accession no. DQ206402) was obtained through the NCBI database (http://blast.ncbi.nlm.nih.gov/Blast.cgi). In brief, RACE PCR and nested PCR were performed using a SMARTer RACE cDNA amplification kit (Clontech, Dalian, China) in accordance with the manufacturer's instruction. At the same time, the gene-specific primers were used to amplify *PEN2*, *PEN3*, and *PEN4* (Supplementary Table 1). The final PCR products of *BigPEN*, *PEN2*, *PEN3*, and *PEN4* were cloned into the pMD-19T cloning vector (Takara, Dalian, China), and 8 positive clones were selected and sequenced.

### Sequence and phylogenetic analysis

The *BigPEN*, *PEN2*, *PEN3*, and *PEN4* sequences were translated conceptually, and the deduced protein was predicted using ExPASy (http://cn.expasy.org/). Similarity analysis was conducted using BLAST (http://blast.ncbi.nlm.nih.gov/Blast.cgi/) and the domain architecture prediction of the proteins was performed using SMART (http://smart.emblheidelberg.de). The neighbor-joining (NJ) phylogenic tree was constructed based on the deduced amino acid sequences of penaeidins by utilizing MEGA 5.0 software (http://www.megasoftware.net/download_form) [26].

### qRT-PCR

qRT-PCR was conducted to detect the mRNA levels of penaeidin genes for tissue distribution assays, pathogenic challenge experiments, or silencing efficiency assays by RNAi *in vivo.* For the tissue distribution assay, shrimp tissues, including eyestalk, epithelium, pyloric ceca, stomach, gill, heart, hepatopancreases, antenna, intestine, and hemocytes, were sampled. Three samples from each tissue were collected from 15 shrimp (5 shrimp pooled together as a sample). For the pathogenic challenge experiments, shrimp were injected with approximately 1 × 10^5^ CFU of *V. parahaemolyticus* or approximately 1 × 10^5^ copies of WSSV particles in 50 μl of PBS. In addition, a control group received a PBS injection. Hemocytes of challenged shrimp were collected at 0, 4, 8, 12, 24, 36, and 48 hours post-injection, and 3 samples at each time point were pooled from 9 shrimp (3 shrimp each sample). The method of total RNA extraction, cDNA synthesis, and qRT-PCR analysis was performed as described [27]. In brief, qRT-PCR analysis was performed in a LightCycler 480 system (Roche, Mannheim, Germany) in a total volume of 10 μl with 1 μl of cDNA diluted 1:10 with ddH_2_O, 5 μl of 2× SYBR Green Master Mix (Takara, Dalian, China), and 250 nM of each primer (Supplementary Table 1). The cycling programme was as follows: 95°C for 2 minutes to activate the polymerase, followed by 40 cycles of 95°C for 15 seconds, 62°C for 1 minute, and 70°C for 1 second. The cycling programme ended at 95°C with 5°C/second calefactive velocity to create the melting curve. The expression level of each gene was calculated relative to the internal control gene *EF-1α* by using the Livak (2^−ΔΔCT^) method.

### Detection of viral loads by ab-PCR

The quantification of WSSV copy number was detected by ab-qPCR. The ab-qPCR was conducted with a forward and reverse primer of *wsv069* (WSSV32678-F/WSSV32753-R), a WSSV single copy gene, and a TaqMan fluorogenic probe (WSSV32706) as described previously [28]. The primers are shown in supplementary Table 1. In brief, a 675-bp DNA amplicon of *wsv069* (32678–32753 in the WSSV genome) (accession no. AF332093.2) was obtained and subcloned into the pMD19-T plasmid. The plasmid pMD19-T containing the 675-bp DNA fragment was used as the internal standard and serially diluted 10-fold to generate a standard curve. The extracted shrimp DNA and the internal standard plasmid were subjected to ab-qPCR. The PCR reaction mixture and cycling conditions were the same as previously described [29]. Each sample from one shrimp was made in three replicates by ab-qPCR. The WSSV genome copies were calculated and normalized to 0.1 μg of shrimp tissue DNA.

### RNAi assays

The dsRNAs, including *BigPEN*, *PEN2*, *PEN3*, *PEN4*, *Dorsal* (accession no. ACZ98167), *Relish* (accession no. ABR14713), and *GFP* (as a control), were generated by *in vitro* transcription with the T7 RiboMAX Express RNAi System kit (Promega, Shanghai, China) using the primers shown in supplementary Table 1. The quality of dsRNA was checked after annealing via gel electrophoresis. The RNAi assay was performed as we described previously [30]. The experimental groups were treated with the injections of dsRNA-BigPEN, dsRNA-PEN2, dsRNA-PEN3, dsRNA-PEN4, dsRNA-Dorsal, or dsRNA-Relish (10 μg dsRNA each shrimp in 50 μl PBS), while the control groups were injected with an equivalent concentration of dsRNA-GFP. Forty-eight hours later, the hemocytes from each group were sampled for qRT-PCR to detect the knockdown efficiency of *BigPEN*, *PEN2*, *PEN3*, *PEN4*, *Dorsal*, and *Relish*. Primer sequences are listed in supplementary Table 1. To investigate the effects of Dorsal or Relish on the expression of penaeidins *in vivo* after WSSV infection, expression of penaeidins in shrimp after receiving *Dorsal* dsRNA or *Relish* dsRNA plus WSSV challenge was detected by qRT-PCR. The mRNA levels of penaeidins were also detected by qRT-PCR with specific primers (Supplementary Table 1).

### Recombinant proteins expression and purification

The coding sequences of *BigPEN-FL* (without N-terminal signal peptide), *BigPEN-R*, and *BigPEN-PEN* were amplified by PCR using corresponding primers (Supplementary Table 1) and subcloned into pET-32a (+) plasmid (Merck Millipore, Darmstadt, Germany). The coding sequences of *PEN2*, *PEN3*, and *PEN4* without N-terminal signal peptides were also subcloned into pET-32a (+) plasmid with specific primers (Supplementary Table 1). Since pET-32a-PEN2 and pET-32a-PEN4 could not be induced by isopropyl β-D-thiogalactoside (IPTG), *PEN2* and *PEN4* were subcloned into the pMAL-c2x plasmid (New England Biolabs, Ipswich, MA, USA) with specific primers (Supplementary Table 1). The coding sequence of *VP19* (NP_477936.1), *VP24* (NP_477524.1), *VP26* (NP_477833.1), *VP28* (NP_477943.1), and *VP16* (NP_477843.1) was cloned into pGEX-4T-1 plasmid (GE Healthcare, Madison, WI, USA) with specific primers (Supplementary Table 1).

The recombinant plasmids of BigPEN-FL, BigPEN-R, BigPEN-PRN, PEN2, PEN3, PEN4, VP19, VP24, VP26, VP28, and VP16 were transformed into *E. coli* Rosetta (DE3) cells (TransGen Biotech, Beijing, China) for expression. After 4 hours of induction with 0.3 mM IPTG at 37°C, cells were pelleted by centrifugation and sonicated for 30 minutes on ice water. The supernatant from the sonicated proteins was purified by using Ni-NTA agarose (Qiagen, Düsseldorf, Germany), GST-resin (GenScript, Nanjing, China), or Amylose Resin (New England BioLabs, Ipswich, MA, USA) according to the manufacturer's instructions. The purified proteins were checked by Coomassie staining or western blot. The concentration of the purified proteins was determined using a BCA protein assay kit (Beyotime Biotechnology, Shanghai, China).

### Antiviral activities assay

To understand the function of penaeidins during WSSV infection, viral titres and survival rates were analyzed after RNAi-mediated knockdown of each penaeidin *in vivo*. For the WSSV challenge experiments, shrimp were injected with 10^5^ copies of WSSV particles by intraperitoneal injection or mock-challenged with PBS as a control 48 hours post-dsRNA injection. Then another 48 hours later, muscle tissues from 8 shrimp were collected. Muscle DNA was extracted with a TIANGEN Marine Animals DNA Kit (TIANGEN, Guangzhou, China) according to the manufacturer's instructions. The quantities of WSSV genome copies were measured by utilizing ab-PCR as described above. The survival rate of each group was recorded every 4 hours. The Mantel–Cox (log-rank *χ*^2^ test) method was used to analyze the differences between groups using GraphPad Prism software (Graphpad, San Diego, CA, USA).

In parallel, a series of rescue experiments were performed to monitor the effect of rPenaeidins on WSSV replication levels *in vivo* or survival rates after the knockdown of *BigPEN*, *PEN2*, *PEN3*, or *PEN4* in shrimp. Each rPenaeidin (10 μg) was first incubated with WSSV for 1 hour, and then the mixture was inoculated into the experimental shrimp. The rMBP and/or rTrx proteins were used as controls. Likewise, viral loads and survival rates were analyzed as above.

### Pull-down assay

Pull-down assays were performed to explore whether the recombinant full-length BigPEN (rBigPEN-FL), rBigPEN-PEN (C-terminal PEN domain of BigPEN), rBigPEN-R (N-terminal RPT domain of BigPEN), rPEN2, rPEN3, or rPEN4 could interact with the main envelope proteins of WSSV (VP19, VP24, VP26, VP28, and VP16). For GST pull-down assays, 100 μl of rBigPEN-FL (1 μg/μl), rBigPEN-PEN (1 μg/μl), rBigPEN-R (1 μg/μl), rPEN2 (1 μg/μl), rPEN3 (1 μg/μl), or rPEN4 (1 μg/μl) was incubated with 100 μl of GST-tagged WSSV protein solutions (1 μg/μl) at 4°C for 2 hours, and then the GST-bind resin was added and incubated for 2 hours at 4°C. The resin was thoroughly washed with PBS. The proteins were eluted with elution buffer (10 mM reduced glutathione and 50 mM Tris-HCl, pH 8.0) and then analyzed using 12.5% SDS-PAGE and western blot analysis. The empty GST-tag vector was used as control. For His pull-down assays, 100 μl of rBigPEN-FL (1 μg/μl), rBigPEN-PEN (1 μg/μl), rBigPEN-R (1 μg/μl), or rPEN3 (1 μg/μl) was incubated with 100 μl WSSV protein solutions (1 μg/μl) at 4°C for 1 hour, and then the Ni-NTA binding resin was added and incubated for 2 hours at 4°C. The resin was thoroughly washed with PBS. The proteins were eluted with elution buffer (0.5 M NaCl, 20 mM Tris–Cl pH 7.4, and 300 mM imidazole) and then analyzed using 12.5% SDS-PAGE and western blot analysis. The Trx vector was used as control. For MBP pull-down assays, 100 μl of rPEN2 and rPEN4 (1 μg/μl) was incubated with 100 μl of WSSV protein solutions (1 μg/μl) at 4°C for 1 hour, and then the MBP-bind resin was added and incubated for 2 hours at 4°C. The resin was thoroughly washed with PBS. The proteins were eluted with elution buffer (20 mM Tris-HCl pH 7.4, 0.2 M NaCl, 1 mM EDTA, 10 mM maltose) and then analyzed using western blot analysis. The MBP-tag empty vector was used as control.

Pull-down assays were also performed to explore whether recombinant PmRab7 or LvRab7 could interact with VP28, and LvpIgR could interact with VP24. The ORF of PmRab7 was synthesized by a sequencing company (Tianyi Huiyuan, Guangzhou, China) and cloned into the pMAL-c2x plasmid using specific primers. The coding sequences of LvRab7 and LvpIgR were cloned into the pMAL-c2x plasmid with specific primers (Supplementary Table 1). The recombinant plasmids were expressed in *E. coli Rosetta* (DE3) cells and purified with Amylose Resin (New England BioLabs, Ipswich, MA, USA) according to the manufacturer's instructions. For GST pull-down assays, 100 μl of rPmRab7 (1 μg/μl) and rLvRab7 (1 μg/μl) was incubated with 100 μl of GST-tagged VP28 protein solutions (1 μg/μl) at 4°C for 1 hour, and 100 μl of rLvpIgR (1 μg/μl) was incubated with 100 μl of GST-tagged VP24 protein solutions (1 μg/μl) at 4°C for 1 hour. The GST-binding resin was then added and incubated for 2 hours at 4°C. The resin was thoroughly washed with PBS. The proteins were eluted with elution buffer (10 mM reduced glutathione and 50 mM Tris-HCl, pH 8.0) and then analyzed via western-blot. The empty GST-tag vector was used as control. For MBP pull-down assays, 100 μl of rPmRab7 (1 μg/μl) and rLvRab7 (1 μg/μl) was incubated with 100 μl of VP28 at 4°C for 1 hour, and 100 μl of rLvpIgR (1 μg/μl) was incubated with 100 μl of VP24 at 4°C for 1 hour. Then the MBP-bind resin was added and incubated for 2 hours at 4°C. The resin was thoroughly washed with PBS. The proteins were eluted with elution buffer (20 mM Tris-HCl pH 7.4, 0.2 M NaCl, 1 mM EDTA, 10 mM maltose) and then analyzed via western blot. The MBP-tag empty vector was used as control.

### Western blotting

The pull-down samples were boiled for 10 minutes, and then the proteins were separated on 12.5% SDS-PAGE gels and then transferred to polyvinylidene difluoride (PVDF) membranes (Merck Millipore, Darmstadt, Germany). After blocking with 3% non-fat milk diluted in TBST buffer (150 mM NaCl, 3 mM EDTA, 0.1% Tween-20, 50 mM Tris-HCl, pH 8.0) for 1 hour, the membrane was incubated with mouse anti-6× His, anti-GST, or anti-MBP for 2 hours at 25°C. After washing in TBST buffer, membranes were incubated for 1 hour at 25°C with a rabbit anti-mouse IgG (H+L)-HRP secondary antibody. Both primary and secondary antibodies were incubated in TBST buffer with 0.5% BSA. Membranes were developed using an enhanced chemiluminescent (ECL) blotting substrate (Thermo Fisher Scientific, Waltham, MA, USA), and the chemiluminescent signal was detected using the 5200 Chemiluminescence Imaging System (Tanon, Shanghai, China).

### Infection-blocking assay *in vitro*

Intact WSSV particles were labelled with fluorescein isothiocyanate (FITC) (1 mg/ml) for 2 hours and then washed with PBS three times. The FITC-labelled WSSV was then mixed with 100 μl of rBigPEN-FL, rBigPEN-PEN, rPEN2, rPEN3, rPEN4, or rTrx (1 mg/ml) and then incubated at 25°C for 1 hour. Hemocytes were collected from healthy *L. vannamei* by centrifugation (1000×*g*, 5 minutes) at 25°C and deposited onto a glass slide in 12-hole microtiter plates for 30 minutes, and then the above virion suspension was added. Subsequently, the glass slices in the wells were washed with PBS three times and fixed with 4% paraformaldehyde at 25°C for 15 minutes. The hemocytes on the glass slides were washed with PBS three times and blocked with 3% bovine serum albumin (dissolved in PBS) for 60 minutes at 25°C. The hemocytes were then incubated with mouse anti-β-actin antibody serum (1:1000 diluted in 2% BSA) (Cell Signaling Technology, Danvers, MA, USA) overnight at 4°C. The hemocytes were then washed with PBS three times and then incubated with anti-mouse IgG (H+L) Alexa Fluor 596 (CST, 1:5000 diluted in 2% BSA) for 60 minutes at 25°C in the dark. After washing with PBS three times, hemocytes were incubated with DAPI 33258 (0.5 mg/ml) (Beyotime, Shanghai, China) for 10 minutes. Finally, the slices were visualized with a confocal laser scanning microscope (Leica TCS-SP5, Wetzlar, Germany). Hemocytes (at least 100 cells/slice) were counted at 1000× magnification [31].

### Colloidal gold labelling and TEM

To investigate whether penaeidins can bind to WSSV virions, rPenaeidins were labelled with 10-nm-diameter gold nanoparticles (Sigma-Aldrich, St. Louis, MO, USA), following a previously reported method [32]. Briefly, the pH of the colloidal gold was adjusted to be at least 0.5 higher than the pI of each penaeidin by using 0.1 N HCl. Then, the saturation isotherm was used to determine the protein/gold ratio for the protein and colloidal gold. The minimal amount of protein necessary to stabilize the gold was determined by adding 1 ml of colloidal gold to 0.1 ml of serial aqueous dilutions of the protein. Approximately 0.1 ml of colloidal gold was added to 100 μg of each penaeidin dissolved in 200 μl of PBS for 10 nM of gold. The solution was left to stand for 10 minutes, and then 1% polyethylene glycol (PEG) was added to a final concentration of 0.04%. The solution was left to stand for 30 minutes and centrifuged for 45 minutes at 50,000×*g*. The supernatant was then removed, and the soft pellet was resuspended in 1.5 ml of PBS containing 0.04% PEG and stored at 4°C. The colloidal gold-labelled penaeidin was diluted 1:10 in PBS containing 0.02% PEG. The empty Trx-His tag protein, BSA, or PBS (as controls) were also labelled with gold nanoparticles. The purified virions were absorbed onto carbon-coated nickel grids and incubated with labelled rBigPEN-FL, rBigPEN-PEN, and rPENs (or rTrx, BSA) for 10 minutes at 25°C. After washing with distilled water three times, the samples were counterstained with 2% sodium phosphotungstate for 1 minute and then observed under a transmission electron microscopy (JEM-100CXII, JEOL, Tokyo, Japan).

### Flow cytometry

Hemocytes were collected from shrimp, washed with PBS three times, and counted using a BD FACSCalibur flow cytometry (BD Biosciences, San Jose, California, USA), and then mixed with 100 μg of rBigPEN-FL, rBigPEN-PEN, rPEN2, rPEN3, and rPEN4 (or rTrx, BSA and PBS as controls) together with 100 μl of FITC-labelled WSSV (10^6^ copies/ml). After incubating for 1 hour at 28°C, hemocytes were detected using cytometry for the signals of FITC and the forward scatter (FSC) values of the cells. An FSC threshold was determined using the detection of free FITC-labelled WSSV in order to eliminate cell debris and WSSV virions. The fluorescence boundary was set based on the detection of auto-fluorescence of the untreated hemocytes. A total of 10,000 events were detected for each sample.

### PcDNA3-pIgR construction and overexpression of WSSV in non-permissive cells

To investigate whether PEN2 protein could interfere with VP24 binding to LvpIgR and block WSSV entry, WSSV DNA was detected in LvpIgR-overexpressed non-permissive cells (HEK293T cells). The ORF of LvpIgR without a stop codon was amplified and cloned into the pcDNA3 plasmid. The primers are listed in supplementary Table 1. HEK293T cells were seeded in a 6-well-plate using Fugene HD Transfection Reagent (Promega Shanghai, China) according to the manufacturer's instructions. Twenty-four hours later, the FITC-labelled WSSV was then mixed with 100 μl of rPEN2 or rTrx (1 mg/ml) and incubated at 25°C for 2 hours. The above virion suspension was then added into the cells and incubated at 37°C for 1 hour. Subsequently, the cells were extensively washed with PBS twice to remove uninfected virus particles, and DNA of the cells was isolated using a TIANGEN Marine Animals DNA Kit (TIANGEN, Guangzhou, China). The isolated DNA was then subjected to qPCR assays to detect the WSSV DNA. The primers used in the qPCR assays are listed in supplementary Table 1.

### Genome walking

The 5′ flanking regulatory regions of *BigPEN* was cloned by genome walking PCR amplification via the GenomeWalker Universal Kit (Clontech, Dalian, China) according to our previous paper [33]. Two pairs of primers AP1/BigPEN-R1 and AP2/BigPEN-R2 were used to perform the first and second round of genome walking PCR amplification. The PCR products were cloned into the pMD-19T vector (Takara, Dalian, China) and sequenced. Primers are listed in supplementary Table 1.

### Dual-luciferase reporter assay

The *L. vannamei* Dorsal and Relish expression vectors (pAc-LvDorsal-V5 and pAc-LvRelsih-V5) were obtained from our previous studies [34,35]. The reporter plasmids, including the promoter regions of *BigPEN*, *PEN2*, *PEN3* or *PEN4*, were cloned using primers (Supplementary Table 1) and then linked into pGL3-Basic (Promega, Shanghai, China) to generate pGL3-BigPEN, pGL3-PEN2, pGL3-PEN3, or pGL3-PEN4, respectively. Two putative NF-κΒ binding sites (κΒ1, _−349_GTGTTTTTCGC_−339_, and κΒ2, _−91_GTGTTTTTTAC_−81_) within the promoter of *BigPEN* were predicted by JASPAR database (http://jaspardev.genereg.net/). Overlap extension PCR using primers (Supplementary Table 1) was performed to construct three mutants of pGL3-κΒ12 with deletions at the κΒ1 site, κΒ2 site, or both sites, and named pGL3-κΒ12, pGL3-κΒ-M1, pGL3-κΒ-M2, and pGL3-κΒ-M12.

Since no permanent shrimp cell line was available, *Drosophila Schneider* 2 (S2) cell line (ATCC CRL 1963) was used instead in order to detect the effects of *L. vannamei* NF-κΒ on the promoters of *L. vannamei* BigPEN, PEN2, PEN3, and PEN4. S2 cells were cultured at 28°C in Schneider's Insect Medium (Sigma-Aldrich, St. Louis, MO, USA) containing 10% fetal bovine serum (Gibco, Grand Island, NY). For dual-luciferase reporter assays, S2 cells were plated into a 96-well plate, and 12 hours later, the cells of each well were transfected with 0.05 μg of firefly luciferase reporter gene plasmids, 0.005 μg pRL-TK renilla luciferase plasmid (Promega Shanghai, China), or 0.05 μg proteins expression plasmids or empty pAc5.1A plasmids (as controls) using the Fugene HD Transfection Reagent (Promega Shanghai, China) according to the user manual. Forty-eight hours post-transfection, the dual-luciferase reporter assays were performed in order to calculate the relative ratios of firefly and renilla luciferase activities using the Dual-Glo Luciferase Assay System kit (Promega Shanghai, China), according to the manufacturer's instructions. All experiments were repeated six times.

### EMSA assay

An EMSA was performed using a Light Shift Chemiluminescent EMSA kit (Thermo Fisher Scientific, Waltham, MA, USA) according to a previously published method [36]. Briefly, the biotin-labelled or unbiotin-labelled probes were designed using the NF-κB binding motif sequence (5′-GTGTTTTTCGC-3′ and 5′-GTGTTTTTTAC-3′). The mutant probe was designed via deleting the NF-κB-binding motif sequence. All of the probes were synthesized by Life Technologies (Shanghai, China), and sequences are listed in supplementary Table 1. Purified rDorsal-RHD (RHD domain of Dorsal) protein (10 μg) was incubated with 20 fmol of the probes for the binding reactions. The reactions were separated on a 5% native PAGE gel, transferred to positively charged nylon membranes (Roche, Germany), and cross-linked by UV light. Then the biotin-labelled DNA on the membrane was detected by chemiluminescence and developed on x-ray film, followed by enhanced chemiluminescence (ECL) visualization (Tanon, Shanghai, China).

### Statistical analysis

All data were presented as means ± SD. Student's *t* test was used to calculate the comparisons between groups of numerical data. For survival rates, data were subjected to statistical analysis using GraphPad Prism software (Graphpad, San Diego, CA, USA) to generate the Kaplan–Meier plot (log-rank *χ*^2^ test).

## Results

### Penaeidins were strongly upregulated *in vivo* after WSSV infection

**Figure 1.**
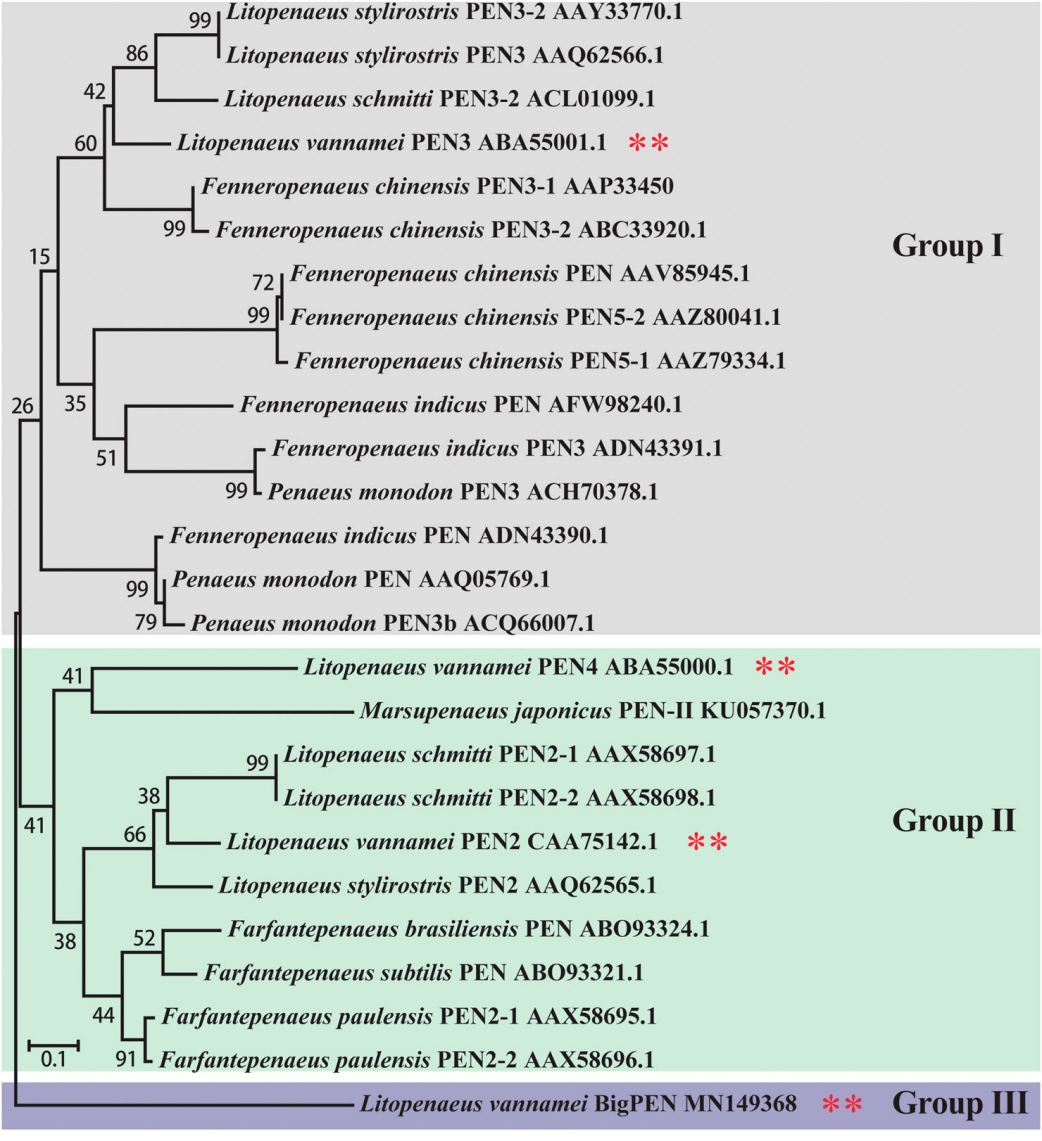
A phylogenetic tree was constructed using amino acid sequences of the PEN domains from different penaeidins. The GenBank accession numbers are shown after scientific names of their species. All reported penaeidins from different penaeid shrimp species can be clustered into three subgroups, and each subgroup contained one or two penaeidins from *Litopenaeus vannamei*. In particular, penaeidin 3 (PEN3) and 5 (PEN5) from *L. vannamei* are clustered in group I. Penaeidin 2 (PEN2) and 4 (PEN4) from *L. vannamei* are clustered in group II. BigPEN from *L. vannamei* is clustered in group III.

**Figure 2.**
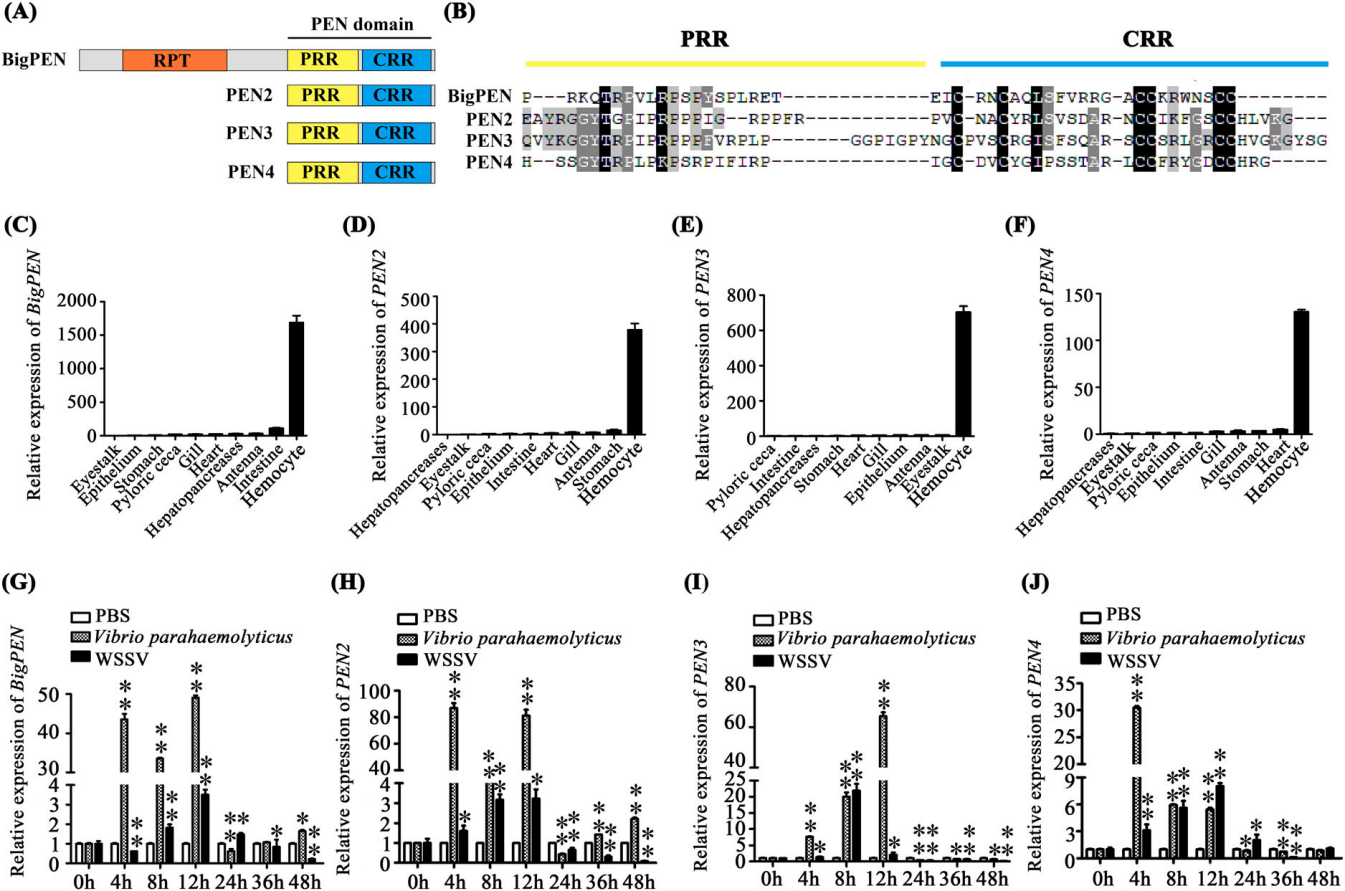
Penaeidins were strongly induced in response to WSSV infection. (A) Architecture diagrams of BigPEN, PEN2, PEN3, and PEN4. The penaeidin domain and different regions are shown with distinct colours. BigPEN contained a conserved PEN domain, and an additional repeat (RPT) region, as compared to PEN2, PEN3, and PEN4. (B) Multiple sequence alignments of the PEN domains, which contained a proline-rich region (PRR) and a cysteine-rich region (CRR), from the four penaeidins. (C–F) Transcriptional levels of (C) *BigPEN*, (D) *PEN2*, (E) *PEN3*, and (F) *PEN4* in different tissues of healthy shrimp were analyzed by quantitative RT-PCR. *L. vannamei EF*-*1α* was used as an internal control, and the data are shown as the mean ± SD of triplicate assays. (G–J) Expression profiles of (G) *BigPEN*, (H) *PEN2*, (I) *PEN3*, and (J) *PEN4* in hemocytes from WSSV- or *V. parahaemolyticus-* or PBS- (as a control) challenged shrimp. Quantitative RT-PCR was performed in triplicate for each sample. Expression values were normalized to those of *EF-1α* using the Livak (2^−ΔΔCT^) method, and the data are provided as the mean ± SD of triplicate assays. The statistical significance was calculated using Student's *t* test (** *P* < 0.01 and * *P* < 0.05).

Penaeidins have been previously identified as AMPs with significant antibacterial and antifungal activities [37]. To explore whether penaeidins have any antiviral roles in the defense against WSSV, we first searched the expressed sequence tag (EST) sequences homologous to known penaeidin proteins from our transcriptome in *L. vannamei* [24] and obtained a new paralog. We cloned the full-length cDNA sequence of the new paralog by using the rapid amplification cDNA ends (RACE)-PCR method. The full-length cDNA sequence of this paralog was 1528 bp, and it encoded a protein of 269 amino acids (accession no. MN149368). We subsequently designated it as BigPEN because it contained an additional repeat (RPT) region and its high molecular weight (29.22 kDa). A total of four penaeidins, including the newly cloned BigPEN and the previously identified PEN2 (DQ206401), PEN3 (DQ206403), and PEN4 (DQ206402) [20], have been identified in *L. vannamei* shrimp and clustered into three major groups (Figure 1). BigPEN has an additional RPT domain as compared with PEN2, PEN3, and PEN4. All four penaeidins contained a conserved PEN domain that consisted of a PRR and CRR (Figure 2A). The PEN domain from each penaeidin contained six conservative cysteines (Figure 2B).

In order to better understand the function of the penaeidin family during WSSV infection, the expression of *BigPEN*, *PEN2*, *PEN3*, and *PEN4* was analyzed to not only determine their tissue distribution in healthy shrimp but also assess their time-course expression patterns in virus-challenged shrimp. By quantitative reverse transcription PCR (qRT-PCR), we observed that the four penaeidins were mainly expressed in hemocytes of naïve (uninfected) shrimp (Figure 2C–F), and thus the hemocyte was used as the target tissue in the following studies. In regard to penaeidins as conventional AMPs, a Gram-negative bacterium, *Vibrio parahaemolyticus,* was chosen as a control pathogen, and the expression levels of penaeidins after WSSV infection were compared to the expression levels of penaeidins after *V. parahaemolyticus* infection. We found that both pathogens markedly induced the expression of all four penaeidins during the early stages of infection in hemocytes (Figure 2G–J). In particular, the upregulation of *BigPEN* and *PEN2* 4–12 hours after *V. parahaemolyticus* infection was increased as compared to WSSV infection. Moreover, the expression of *BigPEN* and *PEN2* displayed different expression profiles 24–48 hours post-infection (hpi), with a slight upregulation and downregulation, respectively (Figure 2G and H). *PEN3* showed increased expression patterns 4–8 hours post-WSSV infection but suppressed expression patterns 12–48 hours post-WSSV infection (Figure 2I). The transcriptional levels of *PEN4* in response to WSSV challenge were sharply upregulated within 4–24 hpi but downregulated at 36 hpi (Figure 2J). Taken together, these results suggested that the induced penaeidins might participate in the immune response against WSSV infection in *L. vannamei*.

### Penaeidins restricted WSSV infection *in vivo*

**Figure 3.**
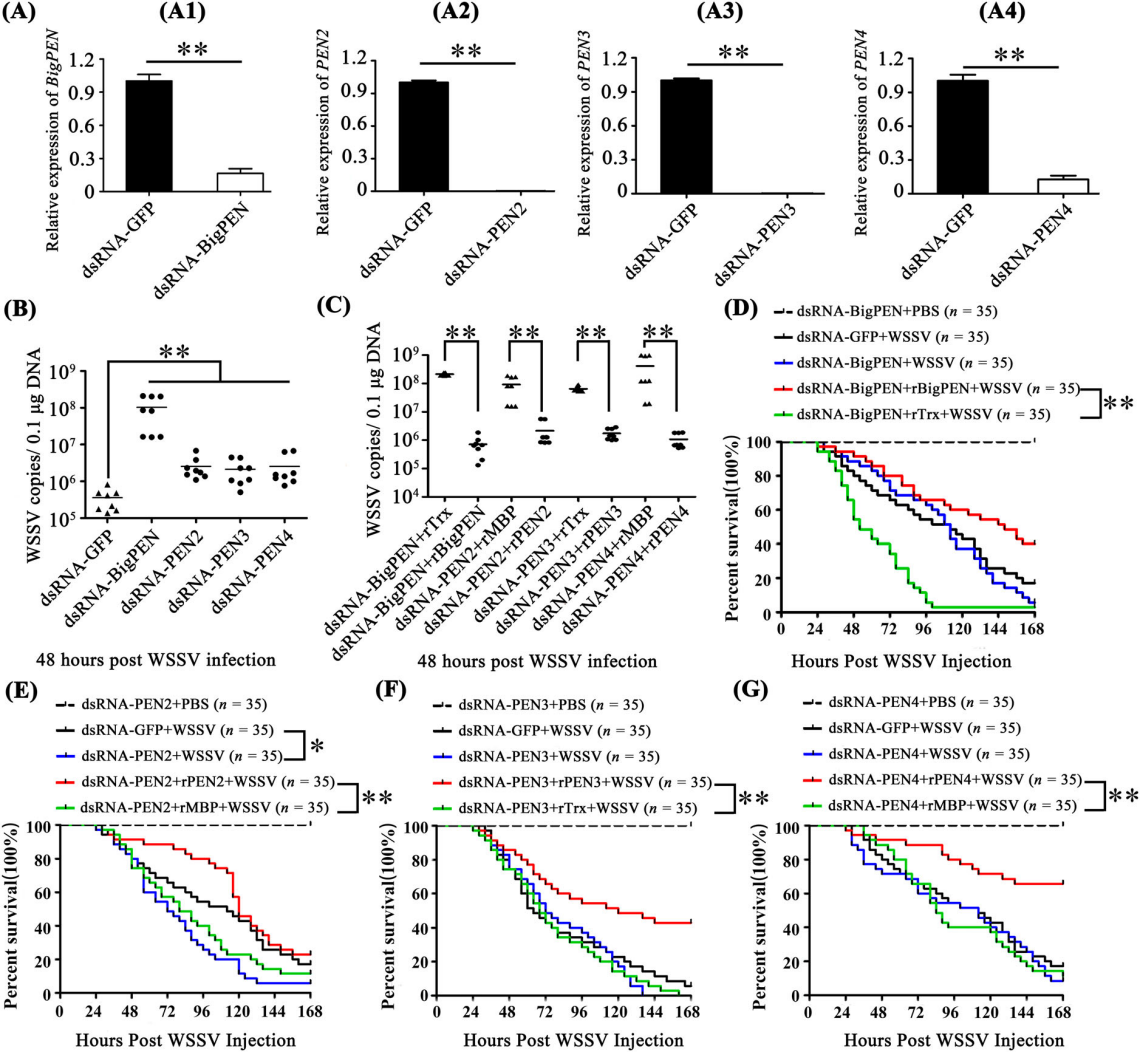
Penaeidins possessed potent antiviral activities against WSSV. (A) Quantitative RT-PCR analysis of the silencing efficiencies of *BigPEN*, *PEN2*, *PEN3,* and *PEN4* in hemocytes. The internal control was *EF-1α*. Samples were taken at 48 hours post-injection and analyzed by quantitative RT-PCR using gene-specific primers for penaeidins or GFP. Differences were analyzed using Student's *t* test (** *P* < 0.01). (B) The quantity of WSSV copies in muscles from each individual shrimp from five different groups was detected by absolute quantitative PCR. After 48 hours of WSSV infection, eight shrimp were used to detect WSSV copies in each group. Differences between the experimental and control groups (GFP dsRNA) were analyzed using Student's *t* test (** *P* < 0.01). (C) The copies of WSSV in the muscles of each individual shrimp from four groups were detected by absolute quantitative PCR. Forty-eight hours post-dsRNA injection, the shrimp were injected with WSSV premixed with purified rBigPEN, rPEN2, rPEN3, or rPEN4. Injections with a similar amount of a mixture of WSSV with Trx or MBP proteins were used as controls. The muscles from each group (8 shrimp) were analyzed by absolute quantitative PCR for WSSV loads at 48 hours post-infection. Differences between the experimental and control groups were analyzed using Student's *t* test (** *P* < 0.01). (D–G) The survival rates of WSSV-infected shrimp with knockdown of penaeidins, including (D) *BigPEN*, (E) *PEN2*, (F) *PEN3*, or (G) *PEN4*. A series of experiments were performed using co-injections of purified recombinant penaeidins to rescue the knockdown of each penaeidin during WSSV infection. The death rate of shrimp was recorded every 4 hours in order to calculate the survival rate by the Kaplan–Meier method (** *P* < 0.01 and * *P* < 0.05). All experiments were performed three times with similar results.

In order to understand the function of penaeidins during WSSV infection, RNA interference (RNAi) combined with injection of recombinant penaeidin proteins (rPenaeidins) was performed *in vivo*. We designed and synthesized different dsRNAs, namely dsRNA-BigPEN, dsRNA-PEN2, dsRNA-PEN3, and dsRNA-PEN4, which specifically targeted *BigPEN*, *PEN2*, *PEN3*, and *PEN4*, respectively. As shown in Figure 3A, the mRNA level of each penaeidin was effectively suppressed by the corresponding gene-specific dsRNA at 48 hours post dsRNA injection. After the knockdown of the penaeidins, the shrimp were infected with WSSV by intramuscular injection, and the viral load (WSSV DNA copies) in each penaeidin-silenced shrimp was determined by absolute quantitative PCR (absolute q-PCR) at 48 hpi. We observed that a greater number of penaeidin-silenced shrimp exhibited higher quantities of viral titres in muscles when compared to the control shrimp (Figure 3B). To further demonstrate the anti-WSSV role of penaeidins, *in vivo* RNAi experiments coupled with rPenaeidins were performed. Our results showed that shrimp co-injected with rPenaeidins and dsRNA had significantly reduced viral replication levels as compared to the control group (Figure 3C). These results strongly indicated that all four penaeidins can inhibit WSSV replication *in vivo*. To investigate whether changes in expression of each penaeidin mediated viral replication levels *in vivo*, survival rate experiments were performed and recorded. We observed that only knockdown of PEN2 resulted in remarkably lower survival rates than the GFP dsRNA control group (*P* = 0.0135 < 0.05) (Figure 3E). Nevertheless, shrimp with knockdown of BigPEN, PEN3, or PEN4 still showed reduced survival rates to some extent, despite no significant difference in the statistical analysis compared with the corresponding control group (Figure 3D, F, and G). It is noteworthy that each rPenaeidin was able to confer shrimp increased resistance to WSSV infection (*P* < 0.01) (Figure 3D–G). This phenomenon could result from the effect of the knockdown of a single penaeidin via RNAi *in vivo*, the function of which might be compensated by other penaeidins or effectors in an unidentified mechanism; however, injection of rPenaeidin not only rescued the silenced penaeidin but also conferred increased protection to shrimp against WSSV infection. In summary, these results convincingly demonstrated that penaeidins are a class of critical antiviral factors against *in vivo* WSSV infection.

### Penaeidins interacted with envelope proteins of WSSV via their PEN domains

**Figure 4.**
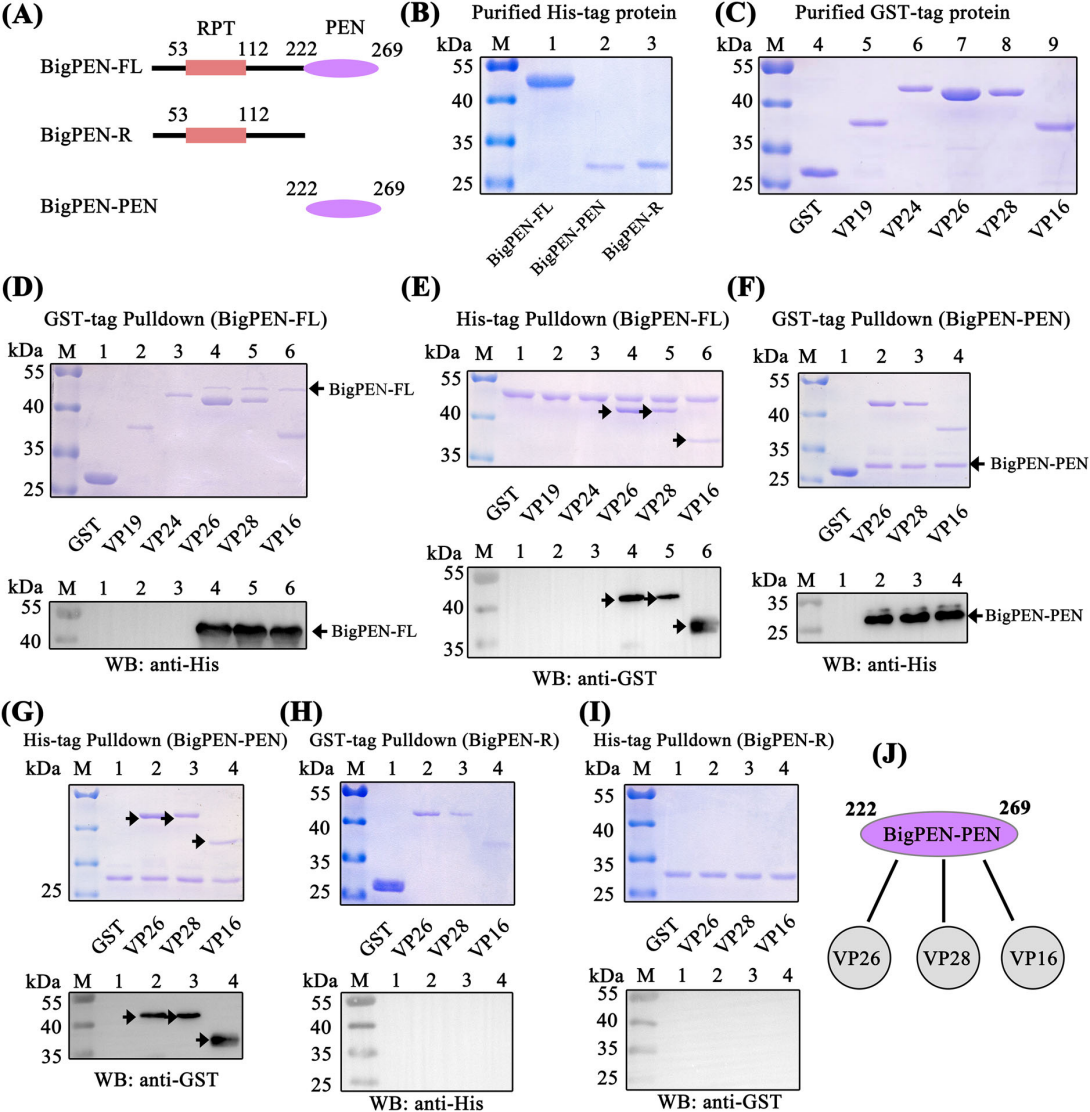
The PEN domain of BigPEN interacted with the envelope proteins of the WSSV. (A) Domain architecture of BigPEN. Three plasmids with a His-tag, including the full-length (BigPEN-FL), the RPT domain (BigPEN-R), and the PEN domain (BigPEN-PEN) of BigPEN, were generated. The light red and purple represent the RPT domain and PEN domain of BigPEN, respectively. (B) Recombinant protein expression and purification of His-tagged BigPEN-FL, BigPEN-R, and BigPEN-PEN. The purified proteins were analyzed using SDS-PAGE and stained with Coomassie blue. (C) Recombination expression and purification of GST, GST-tagged VP19, VP24, VP26, VP28, and VP16. (D–E) Representative images of (D) GST-pulldown and (E) His-pulldown assays to detect the interaction between BigPEN-FL with VP19, VP24, VP26, VP28, and VP16. BigPEN-FL was able to bind VP26, VP28, and VP16. Analysis was performed via staining with Coomassie blue and western blot. A GST-tag protein was used as a control. (F–G) Representative images of (F) GST-pulldown and (G) His-pulldown assays showed that BigPEN-PEN was able to bind to VP26, VP28, and VP16. The results were analyzed via staining with Coomassie blue and western blot. (H–I) Representative images of (H) GST-pulldown and (I) His-pulldown assays demonstrated that the RPT domain of BigPEN (BigPEN-R) did not interact with the five WSSV envelope proteins. (J) Schematic illustration of BigPEN-PEN interacting with VP26, VP28, and VP16. All the experiments were repeated three times.

Direct interaction of antiviral factors with viral proteins has been long postulated as a general antiviral mechanism, especially for enveloped viruses [38,39]. To clarify the possible anti-WSSV mechanism of penaeidins, a pull-down assay was performed to detect whether penaeidin proteins could interact with WSSV envelope proteins. Since BigPEN contained an additional N-terminal RPT domain and conserved C-terminal PEN domain that consisted of a PRR and CRR (Figure 2A), the full-length (BigPEN-FL), RPT domain (BigPEN-R), and C-terminal PEN domain (BigPEN-PEN) with His-tags were expressed and purified (Figure 4A and B). Several envelope proteins of WSSV, including VP19, VP24, VP26, VP28, and VP16 with GST-tags, were also expressed and purified (Figure 4C). In the GST pull-down assays, we observed by Coomassie blue staining that GST-tagged viral proteins, including VP26, VP28, and VP16, precipitated BigPEN-FL (Figure 4D, upper panel, lanes 4−6), and we further confirmed this result by western blotting with a His-tag antibody (Figure 4D, down panel). In the His-tagged BigPEN-FL pull-down assays with five WSSV envelope proteins (GST-tagged), we obtained an identical result showing that BigPEN-FL precipitated VP26, VP28, and VP16 (Figure 4E). Besides, to verify whether rBigPEN-FL was able to interact with VP28 from WSSV infected hemocytes, we performed a pull-down assay by rBigPEN-FL and VP28 (*in vivo*) in hemocytes during WSSV infection. The result showed that rBigPEN could interact with the VP28 from the hemocyte lysates at 24 hours post WSSV infection (Supplementary Figure 1). To further verify which domain of BigPEN was able to interact with WSSV envelope proteins, two separate domains, including BigPEN-R and BigPEN-PEN, were used in pull-down assays. In both of the GST pull-down and His pull-down assays, we observed that BigPEN-PEN, but not BigPEN-R (the RPT domain), was able to interact with VP26, VP28, and VP16 (Figure 4F–I). Collectively, these results strongly demonstrated that the C-terminal PEN domain of BigPEN is able to interact with WSSV envelope proteins, including VP26, VP28, and VP16 (Figure 4J).

**Figure 5.**
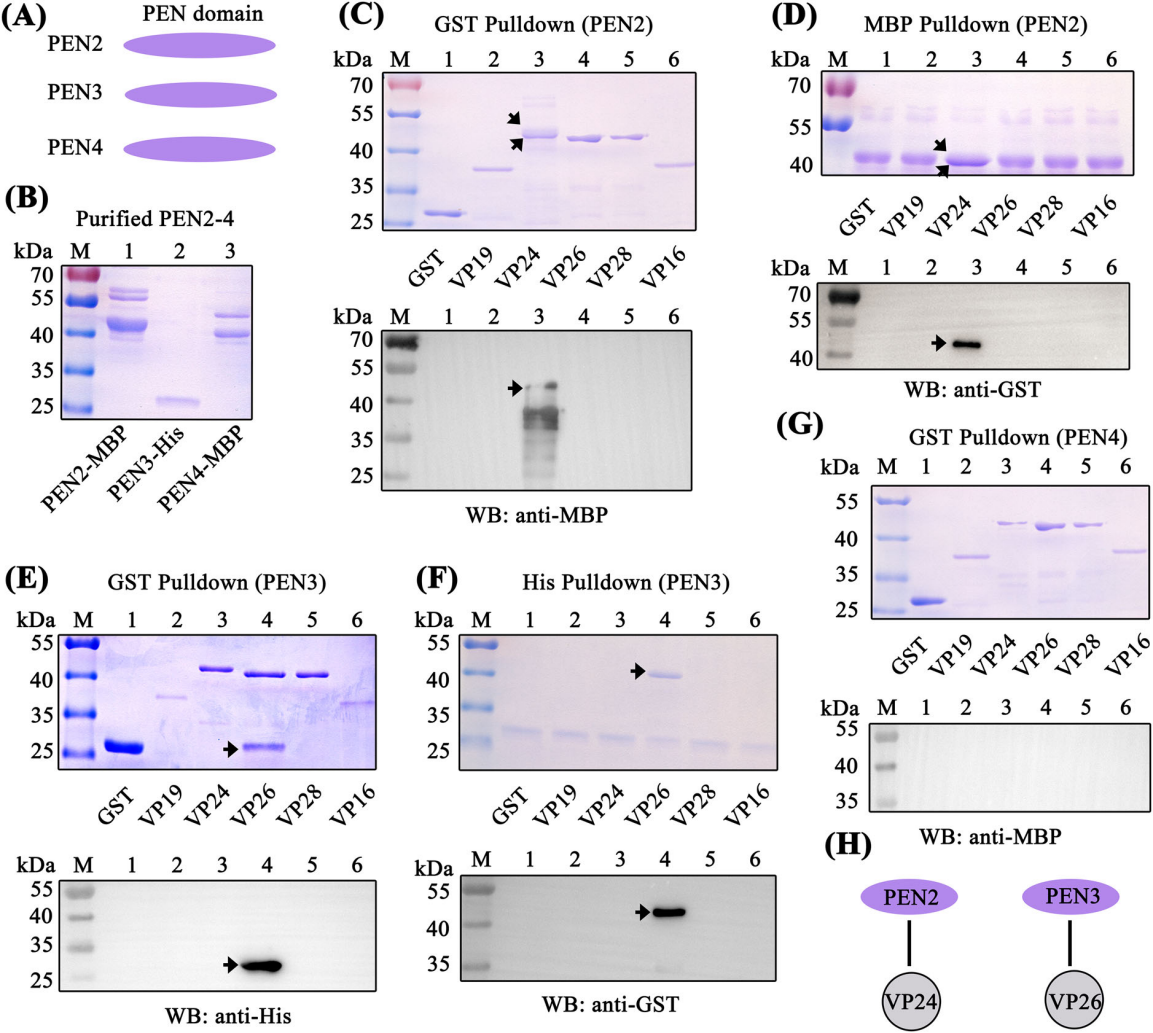
PEN2 and PEN3 interacted with WSSV envelope proteins. (A) Domain architecture of PEN2, PEN3, and PEN4. (B) Recombinant expression and purification of MBP-tagged PEN2 and PEN4 and His-tagged PEN3. (C–D) Both (C) GST-pulldown and (D) MBP-pulldown assays were used to detect the interaction between PEN2 and VP19, VP24, VP26, VP28, or VP16. PEN2 was able to bind VP24, as shown by staining with Coomassie blue and western blot analysis. The up arrow indicated the PEN2-MBP and the down arrow indicated the VP24 in Coomassie blue. (E–F) Both (E) GST-pulldown and (F) His-pulldown assays showed that PEN3 could bind VP26. The interaction was detected by staining with Coomassie blue and western blot analysis. A GST-tag protein was used as a control. (G) A GST-pulldown assay showed that PEN4 was not able to interact with the five WSSV envelope proteins. (H) Schematic illustrations of PEN2 interacting with VP24, and PEN3 interacting with VP26, respectively. All of the experiments were repeated three times.

Since the C-terminal PEN domain of BigPEN showed sequence conservation to the PEN domains of PEN2, PEN3, and PEN4 (Figure 2B), we speculated if the PEN domains of PEN2, PEN3, and PEN4 were also able to interact with some viral envelope proteins. To address this, GST pull-down and His pull-down assays were performed in order to explore the possible interaction between PEN2, PEN3, and PEN4 and the five viral proteins purified above. In contrast to BigPEN-FL protein, PEN2, PEN3, and PEN4 contained only the conserved PEN domains (Figure 2A and Figure 5A). For unknown reasons, the His-tagged PEN2 and PEN4 proteins failed to be expressed, and as such, MBP-tagged proteins were expressed and purified instead (Figure 5B). In the GST pull-down assays, we found that only VP24 was enriched with PEN2 (Figure 5C, upper panel, lane 3), and an identical result was observed by western blot (Figure 5C, down panel, lane 3). Likewise, in the MBP-tagged PEN2 pull-down assays with GST-tagged viral proteins, we observed that PEN2 was able to interact with VP24, but not other tested viral proteins (Figure 5D). By a similar method, PEN3 was demonstrated to specifically bind VP26, but not other tested viral proteins (Figure 5E and F). Unexpectedly, PEN4 did not interact with VP24, VP26, VP28, or VP16 (Figure 5G). Taken together, these results demonstrated that PEN2 was able to interact with VP24, and PEN3 was able to interact with VP26 (Figure 5H).

### Penaeidins inhibited WSSV entry into hemocytes

**Figure 6.**
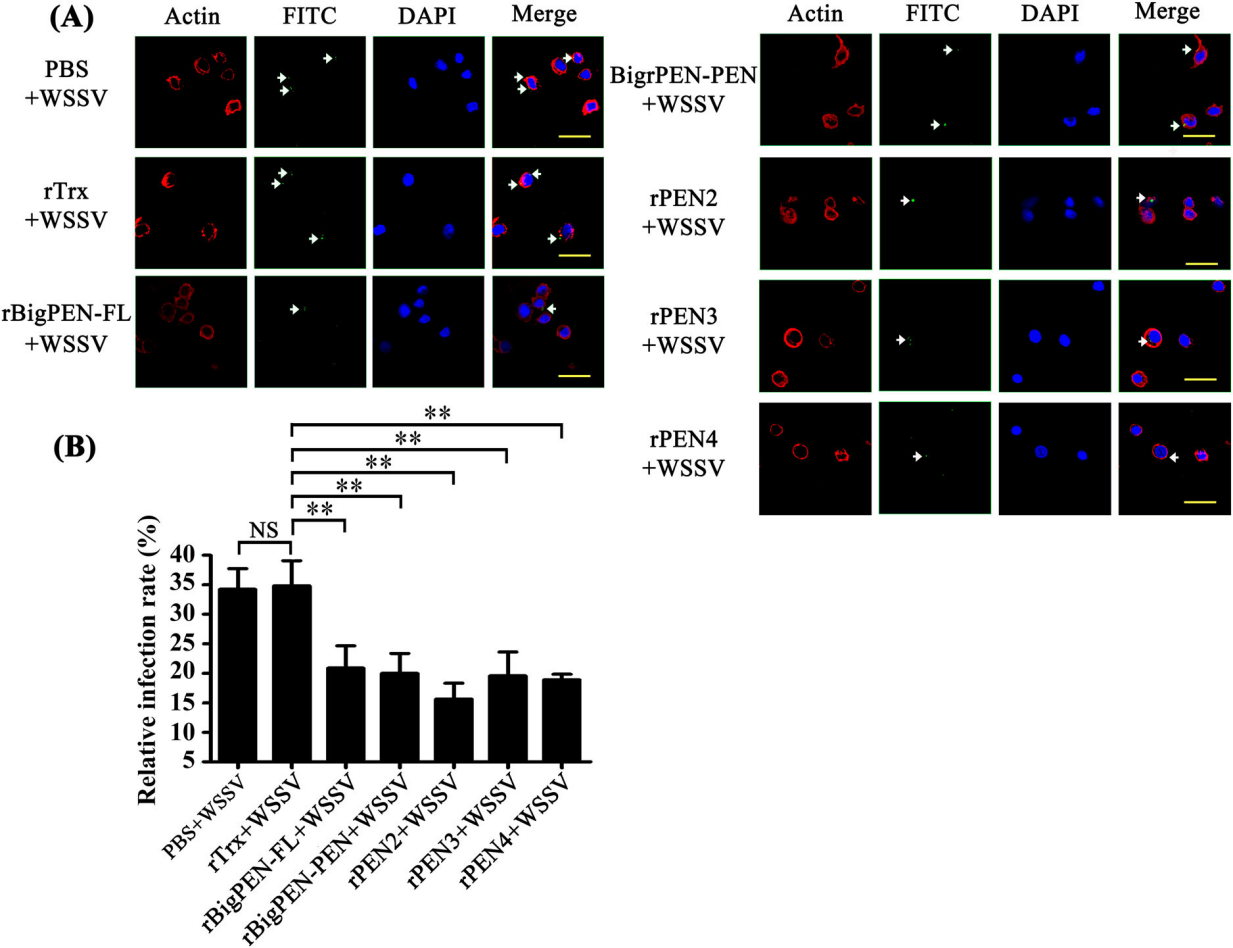
Penaeidins blocked WSSV infection. (A) Penaeidins blocked WSSV entry into hemocytes. Recombinant BigPEN-FL, BigPEN-PEN, PEN2, PEN3, and PEN4 proteins were incubated with FITC-labelled WSSV (green, arrow) and then added to the hemocytes. Cells were fixed, permeabilized, and incubated with primary antibodies directed against actin protein (red, to define the cell shape and cytoplasmic region of the cells), followed by incubation with the appropriate secondary antibodies. Cells were subsequently stained with DAPI (blue, to show the nuclear region of cells) and then observed with a fluorescent microscope. PBS and Trx-tag proteins were used as controls. Scale bar, 25 μm. (B) Statistic analysis of WSSV infection-blocking rates of penaeidins corresponding to (A). All of the data were analyzed statistically by Student's *t* test (** *P* < 0.01, NS, no significant). All experiments were performed three times with similar results.

Successful infection is required for virus entry into host cells [11]. To explore whether the interaction between penaeidins and viral proteins inhibited the entry of WSSV into hemocytes, infection-blocking assays were performed with shrimp hemocytes. FITC was used to label the purified WSSV virions (green, arrow), and red represented actin, which defines the cellular shape and cytoplasmic region of the cell (Figure 6A). The nucleus of the hemocytes was stained with DAPI (blue). The results visually showed that rBigPEN-FL, rBigPEN-PEN, rPEN2, rPEN3, and rPEN4 were able to inhibit WSSV penetration into hemocytes *in vitro* (Figure 6A). The WSSV infection rate of hemocytes was then calculated. PBS and a purified rTrx-tag protein were used as controls. The infection rate of WSSV incubated with PBS or an rTrx-tagged protein was 34.4% and 35.0%, respectively (Figure 6B). Compared to the controls, the WSSV infection rate of hemocytes was remarkably suppressed in the experimental groups by preincubation with rBigPEN-FL (21.0%), rBigPEN-PEN (20.0%), rPEN2 (15.7%), rPEN3 (19.7%), and rPEN4 (19.0%) (Figure 6B). In addition, we found that rBigPEN-R was unable to inhibit WSSV penetration into hemocytes *in vitro* (Supplementary Figure 2A and B). These results strongly suggested that preincubation of penaeidins with WSSV can effectively inhibit virus entry into host hemocytes.

**Figure 7.**
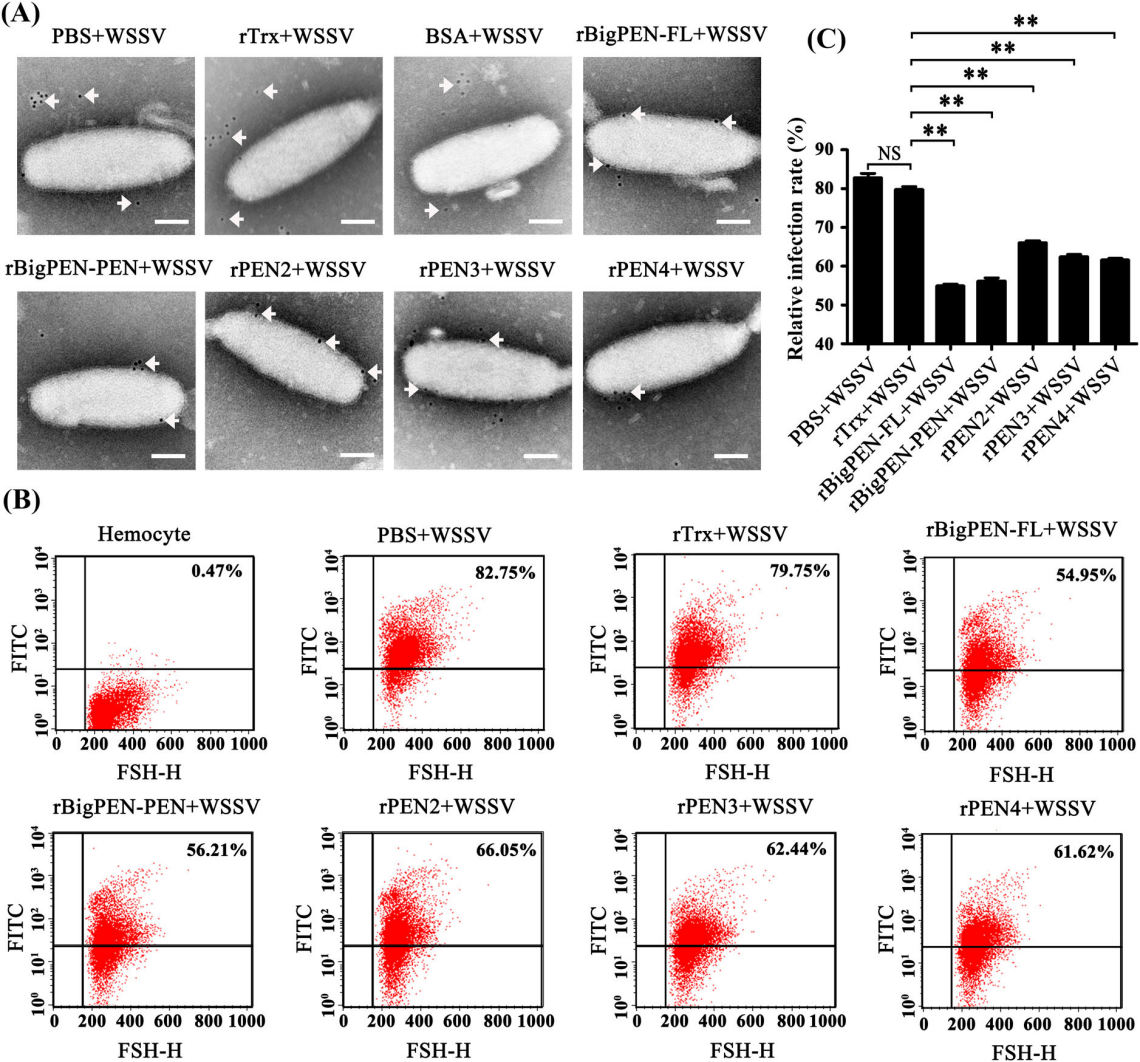
Recombinant penaeidins reduced the infection rate of hemocytes by FITC-labelled WSSV virion. (A) Recombinant penaeidins interacted with the surface of the WSSV virion. Purified recombinant proteins, including BigPEN-FL, BigPEN-PEN, PEN2, PEN3, and PEN4, were labelled with colloidal gold and then incubated with purified WSSV virions. After being stained with phosphotungstic acid, the viral suspension was adsorbed onto carbon-coated nickel grids and observed under transmission electron microscopy (TEM). PBS, Trx-tag protein, and BSA protein were used as controls. Arrows show the locations of colloidal gold-labelled-Trx, -BigPEN, -BigPEN-PEN, -PEN2, -PEN3, or -PEN4. Scale bar: 50 nm. (B) Flow cytometry analysis of the influence of recombinant BigPEN-FL, BigPEN-PEN, PEN2, PEN3, and PEN4 proteins on the infection rate of hemocytes by FITC-labelled WSSV. PBS and a Trx-tag protein were used as controls. Cells were examined by forward scatter (FSC, x-axis), and the infection rate of hemocytes by FITC-labelled WSSV was indicated by intracellular green fluorescence (y-axis). The scatter plots represent one of the three flow cytometric detections. (C) Statistical analysis of infection rates corresponding to (B). All of the data were analyzed statistically by Student's *t* test (** *P* < 0.01). All experiments were performed three times with similar results.

To investigate whether penaeidins were able to interact with WSSV virions, we performed colloidal gold electron microscopy experiments. We observed that each colloidal gold-labelled penaeidin was located on the outer surface of WSSV (Figure 7A), which was consistent with the above results that all penaeidins, except PEN4, were able to interact with one or more envelope proteins of WSSV (Figures 4J and 5H). Although PEN4 failed to interact with the five tested viral envelope proteins, PEN4 was able to interact with the outer surface of WSSV virions, which suggested that PEN4 might have the ability to bind other envelope proteins rather than the five tested proteins in this study. In addition, we observed that colloidal gold-labelled rBigPEN-R was not located on the outer surface of WSSV virions, similar to those of the rTrx-tag and BSA controls (Supplementary Figure 2C). To further confirm the above results, infection-blocking experiments were performed using flow cytometry. The gates were created based on the hemocytes, and R1 is representative of intact hemocytes (Supplementary Figure 2D). We observed that each recombinant penaeidin significantly reduced the WSSV infection rate of hemocytes (Figure 7B). Specifically, the WSSV infection rate of hemocytes preincubated with rBigPEN-FL (54.95%), rBigPEN-PEN (56.21%), rPEN2 (66.05%), rPEN3 (62.44%), and rPEN4 (61.62%) was significantly reduced compared to those of the rTrx tag (79.75%) and PBS (82.75%) controls (*P* < 0.01) (Figure 7C). Furthermore, we observed that rBigPEN-R did not reduce the WSSV infection rate of hemocytes (Supplementary Figure 2E). The WSSV infection rate of hemocytes preincubated with rBigPEN-R (78.77%) was not significantly different from that of the rTrx-tagged protein (79.81%) and BSA (79.75%) controls (Supplementary Figure 2F). In summary, these results convincingly showed that penaeidins were able to inhibit WSSV entry into host cells, perhaps by interacting with viral envelope proteins.

### PEN2 interfered with VP24 binding to LvpIgR, a host entry receptor for WSSV

**Figure 8.**
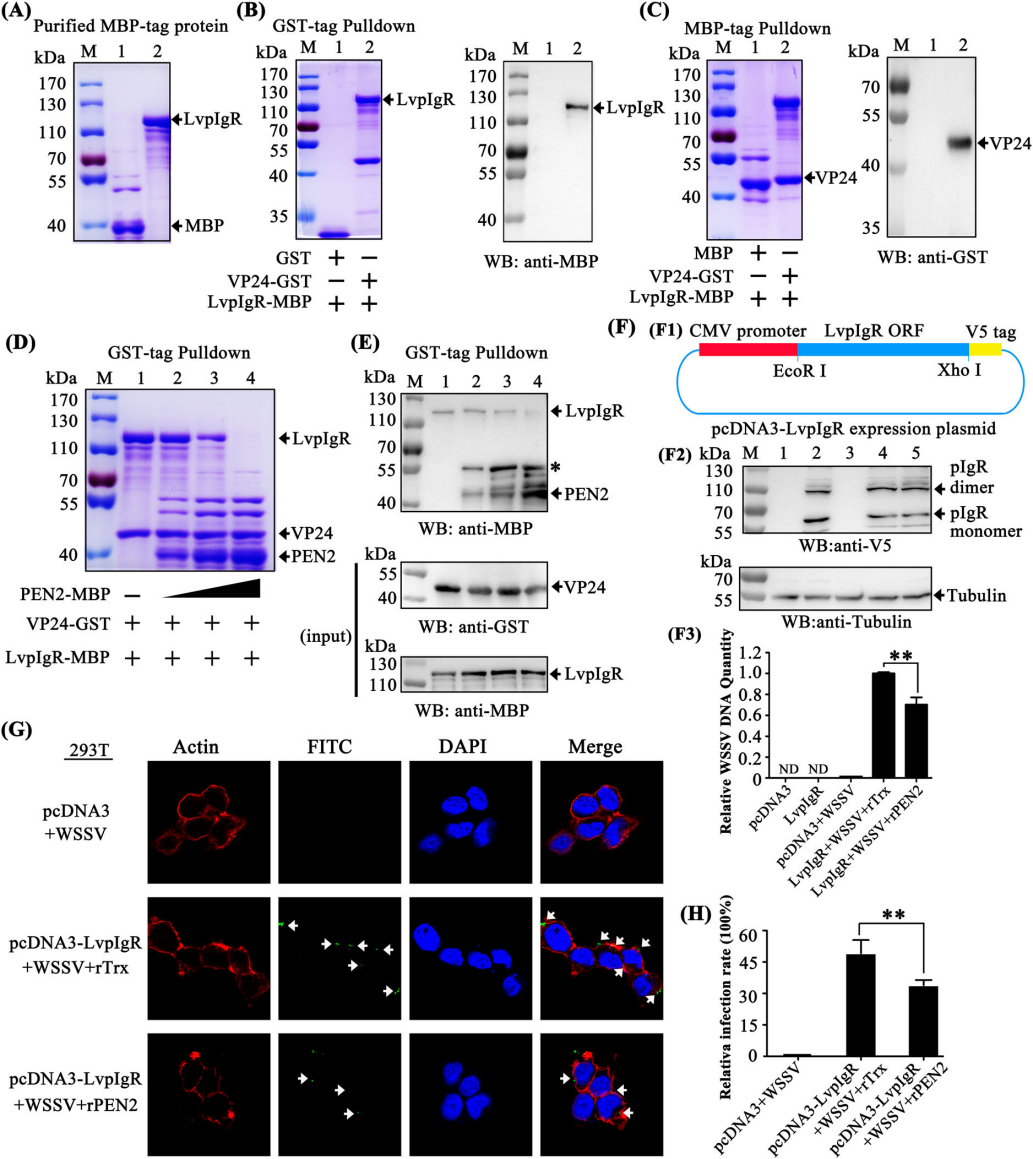
PEN2 interfered with VP24 binding to LvpIgR and attenuated WSSV entry into non-permissive cells (HEK293T) transfected with LvpIgR. (A) Recombinant expression and purification of MBP-tagged LvpIgR (the polymeric immunoglobulin receptor (pIgR) from *L. vannamei*). (B–C) The interaction between VP24 and LvpIgR was detected via (B) GST-pulldown and (C) MBP-pulldown assays. VP24 was able to bind LvpIgR, as shown by staining with Coomassie blue and western blot analysis. (D–E) PEN2 competitively bound VP24 to release it from pIgR, as shown by (D) Coomassie blue staining and (E) Western blot analysis of a GST-pulldown assay, the asterisk indicated nonspecific bands. (F) PEN2 attenuated WSSV entry into non-permissive cells (HEK293T) transfected with LvpIgR. (F1) pcDNA3-pIgR map. (F2) Western blot analysis of LvpIgR expression in HEK293T cells. Lane 1, uninfected HEK293T cells transfected with pcDNA3 empty plasmid; lane 2, uninfected HEK293T cells transfected with pcDNA3-pIgR plasmid; lane 3, HEK293T cells transfected with pcDNA3 empty plasmids infected with WSSV; line 4, HEK293T cells transfected with pcDNA3-pIgR plasmid infected with WSSV premixed with the rTrx protein; lane 5, HEK293T cells transfected with pcDNA3-pIgR plasmid infected with WSSV premixed with the rPEN2 protein. (F3) qPCR analysis of WSSV DNA in WSSV-infected HEK293T cells. The qPCR results are presented relative to genomic DNA. ND: Not Detectable. (G) PEN2 attenuated WSSV entry into entry into non-permissive cells (HEK293T) by fluorescence microscopy. HEK293T cells transfected with pcDNA3-pIgR plasmid. After 24 hours, recombinant PEN2 was first incubated with the FITC-labelled WSSV (green) and then added into the HEK293T cells. Cells were fixed, permeabilized, and incubated with primary antibodies directed against actin protein (red), followed by incubation with appropriate secondary antibodies. Cells were subsequently stained with DAPI (blue) and then observed with a fluorescent microscope. PBS and a Trx-tag protein were used as controls. Scale bar, 25 μm. (H) Statistic analysis of WSSV infection-blocking rates of penaeidins corresponding to (G). All the experiments were repeated three times with similar results.

The preceding studies showed that penaeidins block WSSV entry into hemocytes, which suggests that penaeidins interfere with the infection process. Viral receptors play significant roles in the initial step of viral entry and are potential targets for penaeidins. A recent study identified that the polymeric immunoglobulin receptor (pIgR) from *M. japonicus* shrimp is a host entry receptor for WSSV, and the envelope protein VP24 is the receptor-binding protein [14]. Thus, we investigated if PEN2 interfered with VP24 binding to the pIgR homolog from *L. vannamei* (LvpIgR) (accession no. MN164612), resulting in an inhibition of the infection process. To determine this, we first investigated whether LvpIgR served as a host entry receptor for WSSV in *L. vannamei* shrimp. We cloned the full length of LvpIgR from our transcriptome data and found that LvpIgR was highly homologous to MjpIgR with sequence identity up to 91% (data not shown). MBP-tagged LvpIgR was expressed and purified (Figure 8A). Using GST pull-down assays, we observed that GST-tagged VP24 interacted with MBP-tagged LvpIgR by Coomassie blue staining (Figure 8B left panel, lane 2), and we further confirmed this result by western blot analysis with an MBP tag antibody (Figure 8B right panel, lane 2). In the MBP-tagged LvpIgR pull-down assays with VP24, we obtained an identical result showing that LvpIgR interacted with VP24 by Coomassie blue staining (Figure 8C left panel, lane 2), and we further confirmed this result by western blot analysis with GST tag antibody (Figure 8C right panel, lane 2). These results indicated that not only VP24 interacted with LvpIgR but also LvpIgR may serve as the host entry receptor for WSSV in *L. vananmei* shrimp.

In the previous result, we found that PEN2 could interact with VP24 (Figure 5H). Next, we explored whether PEN2 interfered with VP24 binding to LvpIgR. The GST-tagged LvpIgR-SC (the extracellular domains of LvpIgR) was expressed and purified (Supplementary Figure 3A). Using MBP-tag pulldown assays, we verified that PEN2 did not interact with LvpIgR-SC by Coomassie blue staining (Supplementary Figure 3B) and western blot analysis (Supplementary Figure 3C). We then performed a GST pulldown experiment using three recombinantly expressed proteins and found that VP24 interacted with LvpIgR. Furthermore, when PEN2 was present in different amounts, the binding between VP24 and LvpIgR was affected – as the concentration of PEN2 increased, the binding of LvpIgR to VP24 decreased (Figure 8D). We further confirmed this result by western blot, and the VP24 and LvpIgR inputs were also assessed by western blot (Figure 8E). These results demonstrated that PEN2 interfered with VP24 binding to LvpIgR, thereby disrupting the interaction between VP24 and LvpIgR.

To address whether PEN2 inhibited WSSV internalization by interfering with the interaction between VP24 and LvpIgR, we assessed the level of WSSV entry into non-permissive cells (HEK293T) with ectopic expression of LvpIgR. The ORF of LvpIgR without a stop codon was constructed in the plasmid pcDNA3 (Invitrogen) to generate pcDNA3-pIgR that was used to express V5-tagged LvpIgR in HEK293T cells (Figure 8F1). The cells were infected with WSSV premixed with the purified rPEN2 or rTrx protein for one hour or remained uninfected. After WSSV infection, the DNA of the cells was isolated and subjected to quantitative PCR assay to detect WSSV DNA. The expression of LvpIgR in HEK293T cells was confirmed by western blot analysis after transfection (Figure 8F2), which showed that LvpIgR exhibited some homo-oligomerization properties, similar to a previous report [14]. Viral DNA was detected in the pcDNA3-pIgR transfected HEK293T cells, but not in the cells transfected with the empty vector (Figure 8F3), which confirmed that LvpIgR was a bona fide receptor for WSSV internalization. Compared to the control, the relative quantity of WSSV DNA was remarkably reduced in the pcDNA3-pIgR-transfected HEK293T cells infected with WSSV and preincubated with PEN2 (*P* < 0.01) (Figure 8F3). To further verify that PEN2 blocked WSSV entry into non-permissive cells (HEK293T) by interfering with the interaction between VP24 and LvpIgR, infection-blocking assays were performed. The results showed that rPEN2 was able to inhibit WSSV entry into non-permissive cells (HEK293T) transfected with LvpIgR *in vitro* (Figure 8G). The WSSV infection rate of HEK293T cells was then calculated. Purified rTrx was used as a control. The WSSV infection rate of cells preincubated with rTrx was 49.08% (control). Compared to the control, the infection rate of HEK293T cells was reduced in the experimental group infected with WSSV and preincubated with rPEN2 (33.87%; *P* < 0.01) (Figure 8H). Taken together, these results demonstrated that PEN2 protein interfered with VP24 binding to LvpIgR, which inhibited WSSV entry.

### BigPEN interfered with VP28 binding to LvRab7, a key regulator for WSSV endocytosis

**Figure 9.**
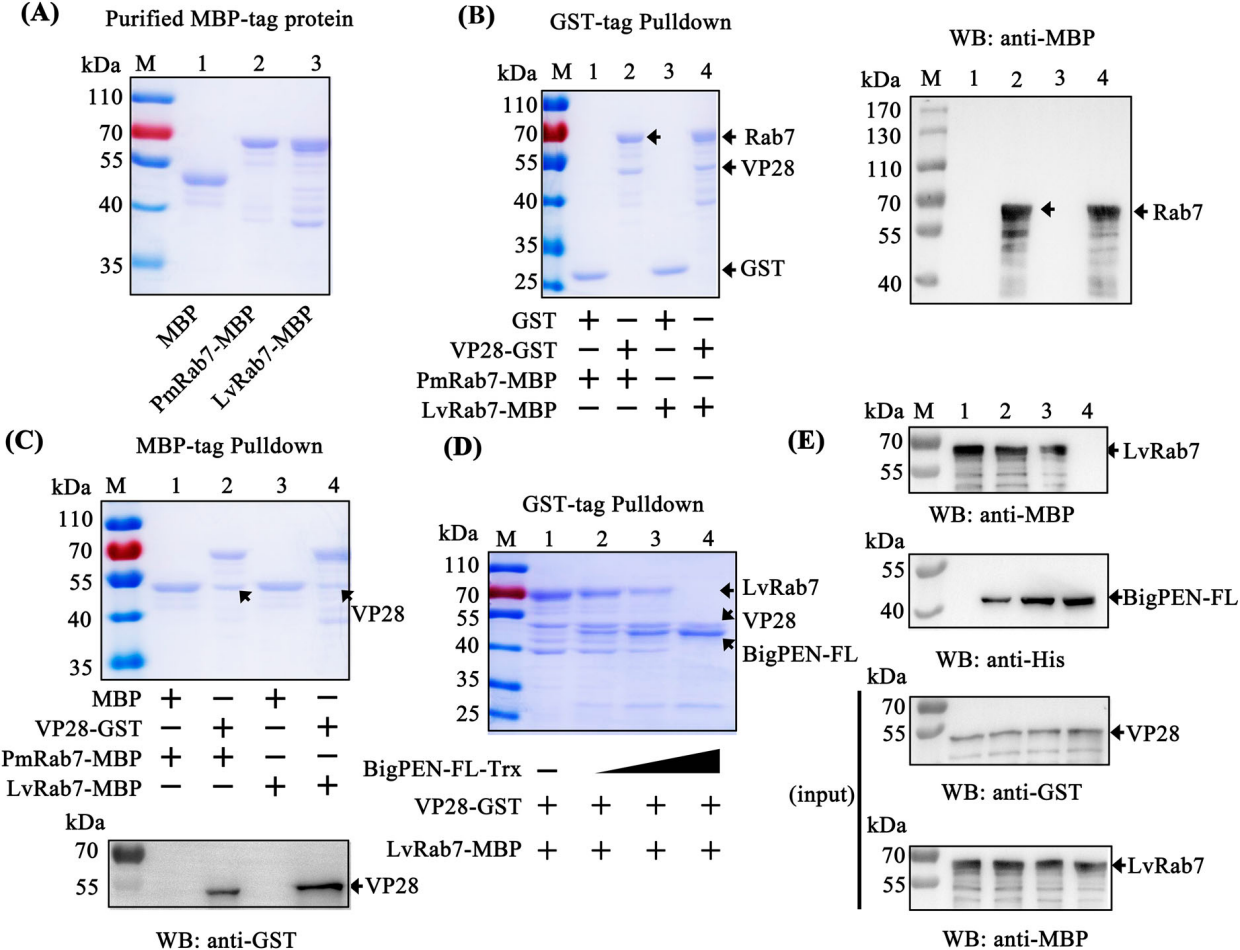
BigPEN interfered with VP28 in binding LvRab7. (A) Recombinant expression and purification of MBP-tagged PmRab7 and LvRab7. (B–C) The interaction between VP28 with PmRab7 or LvRab7 was detected via (B) GST-pulldown and (C) MBP-pulldown assays. VP28 was able to bind PmRab7 or LvRab7, as shown by staining with Coomassie blue and western blot analysis. (D–E) BigPEN interfered with the VP28-LvRab7 interaction as shown by (D) Coomassie blue staining and (E) western blot analysis of a GST-pulldown assay. All of the experiments were repeated three times with similar results.

In general, viral entry starts with binding to the cellular receptor, followed by endocytosis. After binding to the cellular receptor, such as pIgR, WSSV has been shown to use endocytosis as a route of entry into host cells [14]. Previous studies have shown that Rab7 GTPases are key regulatory proteins involved in endocytosis [40]. The *P. monodon* Rab7 (PmRab7, accession no. HQ128578) has been shown to interact with WSSV protein VP28, which is beneficial for WSSV infection [13]. Therefore, we cloned the full-length *L. vannamei* Rab7 (LvRab7, accession no. FJ811529) protein and found that LvRab7 shares 100% sequence identity to PmRab7 (data not shown), which suggests that LvRab7 might also be able to interact with VP28. To address this, MBP-tagged PmRab7 (as a control) and LvRab7 were expressed and purified (Figure 9A). Using GST pull-down assays, we observed that GST-tagged viral protein VP28 interacted with MBP-tagged PmRab7 and LvRab7 (Figure 9B left panel, lanes 2 and 4), and further confirmed this result by western blot analysis using an MBP-tag antibody (Figure 9B right panel). In addition, Coomassie blue staining also demonstrated that MBP-tagged PmRab7 and LvRab7 were able to pull down VP28 (Figure 9C upper panel, lanes 2 and 4). We further confirmed this result by western blot analysis using a GST-tag antibody (Figure 9C down panel). These results confirmed the interaction between WSSV VP28 and PmRab7, as previously reported [13], as well as the binding of WSSV protein VP28 with LvRab7.

In the previous result, we found that BigPEN-FL could interact with VP28 (Figure 4D and 4J). In order to explore whether BigPEN-FL could interfere with VP28 binding to LvRab7, we performed a competitive protein binding assays via a GST pulldown with three recombinantly expressed proteins. Using His-tag pulldown assays, we first verified by Coomassie blue staining (Supplementary Figure 3D) and western blot analysis (Supplementary Figure 3E) that BigPEN-FL did not interact with PmRab7 or LvRab7. The GST pulldown experiment using three recombinantly expressed proteins showed that LvRab7 could interact with VP28, and when BigPEN-FL was present in different amounts, the binding between LvRab7 and VP28 was affected, such that increased concentrations of BigPEN-FL resulted in decreased LvRab7-VP28 binding (Figure 9D). This result was further confirmed by western blot analysis, and the VP28 and LvRab7 inputs were also detected by western blot (Figure 9E). Taken together, these results clearly demonstrated that BigPEN-FL interfered with VP28 binding to LvRab7.

### Penaeidins were regulated by conserved NF-κB pathways

**Figure 10.**
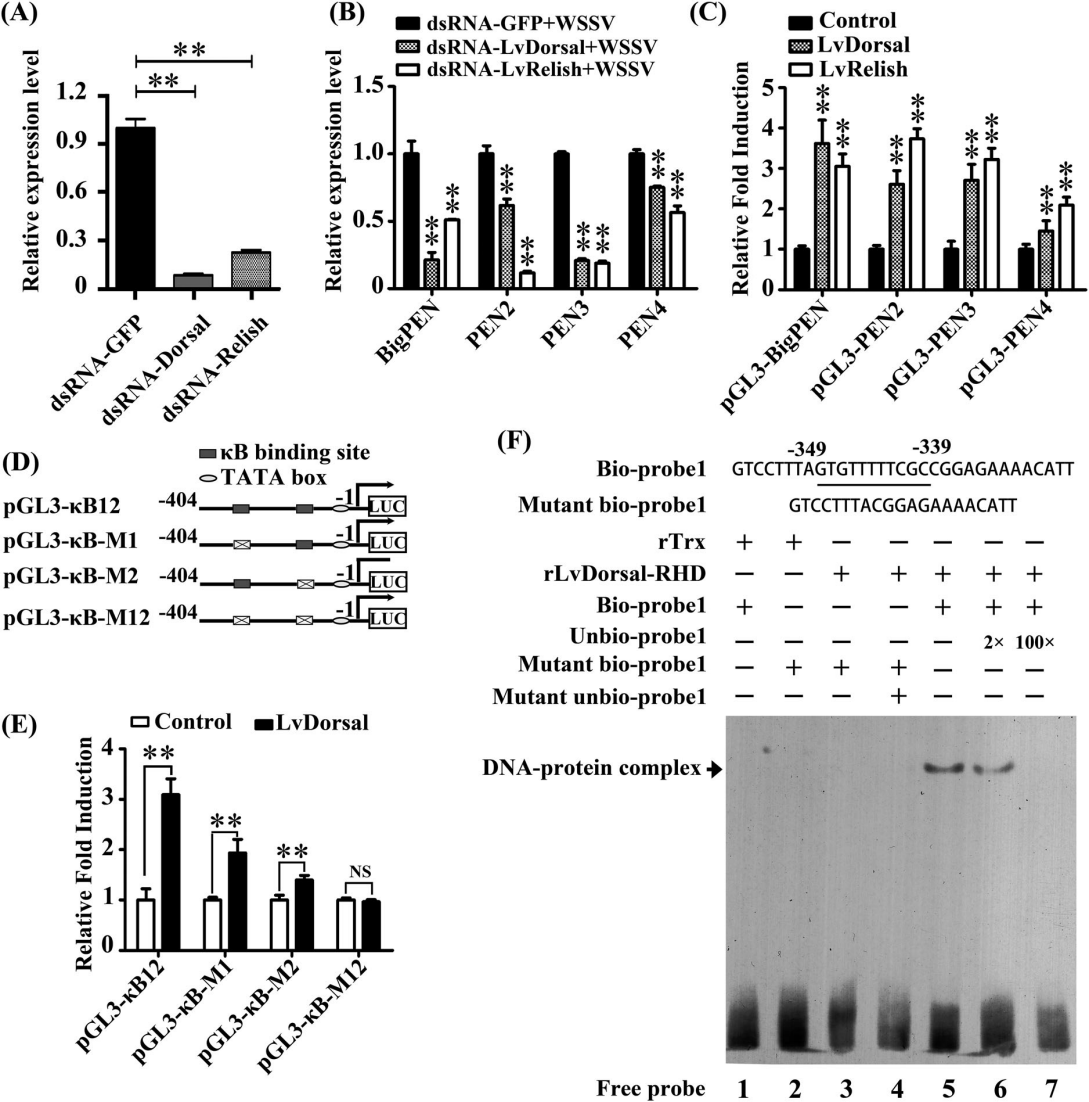
Penaeidins were regulated by NF-κB pathways. (A) Effective knockdown for *Dorsal* and *Relish* in hemocytes by dsRNA was confirmed by qRT-PCR. Differences were analyzed using Student's *t* test (** *P* < 0.01). (B) The mRNA levels of *BigPEN, PEN2, PEN3,* and *PEN4* in the hemocytes of Dorsal- and Relish-silenced shrimp at 48 hours post-WSSV infection. The statistical significance was calculated using Student's *t* test, ** *P* < 0.01. (C) Dual-luciferase reporter assays were performed to analyze the effects of the overexpression of Dorsal and Relish on the promoter activities of *BigPEN, PEN2, PEN3,* and *PEN4* in *Drosophila* S2 cells. All data are representative of three independent experiments. The value of cells transfected with an empty plasmid (pAc5.1/V5-His A), which were used as a control, was set as 1.0. The bars indicate the mean ± SD of the relative luciferase activities (*n* = 3). The statistical significance was calculated using Student's *t* test (** *P* < 0.01). (D) Schematic diagram of the promoter regions of *BigPEN* in the luciferase reporter gene constructs. (E) Dual-luciferase reporter assays were performed to analyze the effects of the overexpression of Dorsal on the promoter activities of *BigPEN* with or without mutated NF-κB binding motif(s). The bars indicate mean values ± S.D. of the luciferase activity (*n* = 3). Statistical significance was determined by Student's *t* test (** *P* < 0.01). (F) Dorsal interacted with the NF-κB binding motif of *BigPEN in vitro*. An EMSA was performed using biotin-labelled (Bio-) or unlabeled (Unbio-) probes containing or not containing the NF-κB binding motif of *BigPEN*. Biotin-labelled or mutated biotin-labelled dsDNA probes were incubated with 10 μg of purified rDorsal-RHD protein. Unlabeled probe was added to compete with binding, and an rTrx protein was used as a control. All experiments were performed three times with similar results.

In invertebrates, the transcriptional expression of AMPs is commonly regulated by conserved innate immune signalling pathways, such as Toll, IMD, and JAK-STAT pathways [41–43]. In the shrimp *L. vannamei*, Dorsal and Relish (NF-κB), the downstream transcription factors of Toll and IMD signalling pathways, respectively, were regarded to be the major factors that directly induce the production of AMPs in response to infection [44]. To explore whether the expression of penaeidins was regulated by Dorsal and Relish, an RNAi *in vivo* experiment was performed. By quantitative RT–PCR analysis, the mRNA levels of *Dorsal* and *Relish* were effectively suppressed by corresponding dsRNAs (*P* < 0.01) (Figure 10A). We then observed that the silencing of Dorsal or Relish resulted in varying degrees of downregulation in the transcript levels of *BigPEN*, *PEN2*, *PEN3*, and *PEN4* under WSSV challenge *in vivo* (*P* < 0.01) (Figure 10B). To address whether Dorsal and Relish were able to regulate the expression of penaeidins *in vitro*, a dual-luciferase reporter assay and electrophoretic mobility shift assay (EMSA) were performed. We first obtained the promoter regions of the four penaeidins, including *BigPEN, PEN2, PEN3,* and *PEN4,* by a genome walking method (Supplementary data 1) and then cloned them into the pGL3-Basic vectors. We observed that overexpression of *L. vannamei* Dorsal or Relish could significantly induce the promoter activities of all four penaeidins in *Drosophila* S2 cells (Figure 10C). The above results suggested that both Dorsal and Relish were able to induce the expression of all four penaeidins *in vivo* and *in vitro* (*P* < 0.01). Subsequently, BigPEN was chosen to further confirm these results in detail. We analyzed the 5′ flanking regulatory region of *BigPEN* and found that it contained two conserved κB motifs located at −349 to −339 (κB1, GTGTTTTTCGC) and −91 to −81 (κB2, GTGTTTTTTAC) (Figure 10D). Four vectors, including the wild-type promoter region, termed pGL3-κB12, pGL3-κB-M1, pGL3-κB-M2, and pGL3-κB-M12 vectors, with a deletion mutant of one or both κB sites (Figure 10D), were constructed to perform dual-luciferase reporter assays. We found that the promoter activities of pGL3-κB12, pGL3-κB-M1, and pGL3-κB-M2 were upregulated by *L. vannamei* Dorsal overexpressed in S2 cells with 3.09-, 1.93-, 1.40-fold increases, respectively, whereas the activity of pGL3-κB-M12 was not upregulated (Figure 10E). These results suggested that Dorsal was able to interact with the conserved κB sites within the promoter region of *BigPEN*. To address this, an EMSA was performed using the purified 6His-tagged RHD domain of Dorsal protein (rDorsal-RHD) expressed in *E. coli* cells. As shown in Figure 10F, *L. vannamei* rDorsal-RHD, but not the control rTrx, effectively retarded the mobility of the bio-labelled probe 1 (Line 5). We further observed that the DNA/protein complex was faintly reduced by the competitive 2× unlabeled probe 1 but markedly reduced by the competitive 100× unlabeled probe 1 (Figure 10F, lines 6 and 7). In addition, rDorsal-RHD was not able to retard the mobility of the mutant bio-labelled probe 1 (Figure 10F, line 3), indicating the specificity of interaction between rDorsal-RHD and probe 1. Taken together, the results suggested that NF-κB transcription factors (Dorsal and Relish) participated in the transcriptional expression of penaeidins in response to WSSV infection.

## Discussion

In invertebrates, some AMPs are identified as viral responsive effectors; however, the molecular mechanism underlying the antiviral activities of AMPs is poorly understood. For example, two *Drosophila* AMPs, attC and dptB, have been demonstrated to restrict Sindbis virus (SINV) infection, but the actual antiviral mechanism is still unknown [45]. Herein, four penaeidins identified in *L. vannamei,* including BigPEN, PEN2, PEN3, and PEN4, were assessed in their potential anti-WSSV activities. The critical role of penaeidins during an innate anti-WSSV response was demonstrated by using RNAi *in vivo* to silence each individual penaeidin. We observed that knockdown of each penaeidin resulted in higher viral loads, whereas each purified penaeidin could effectively confer protection to shrimp against the WSSV. In addition, we identified that the antiviral mechanism of penaeidins involved blocking viral internalization and disrupting the process of infection. In summary, we show for the first time that penaeidins are a novel class of anti-WSSV effectors in shrimp.

The production of antiviral effectors represents a major host defense mechanism against viruses in invertebrates, including shrimp [46]. Since invertebrates lack an adaptive immune response, it is rationalized that identifying and characterizing novel antiviral molecules may shed light onto the innate antiviral response in shrimp. In this study, we focused our attention on penaeidins due to the following aspects: (i) penaeidins are a type of AMP that are abundant in penaeid shrimps; (ii) the four penaeidins from *L. vannamei* were significantly induced by WSSV infection (Figure 1A); and (iii) the antiviral activities of penaeidins against the WSSV have not been previously uncovered. In fact, similar to our observations, previous studies have also shown that *L. vannamei* PEN2, PEN3, and PEN4 were strongly upregulated during the early stage of WSSV infection [47], indicating that penaeidins play an important role in the innate antiviral response during early WSSV infection to prevent WSSV dissemination. In order to comprehensively analyze the entire family of penaeidins during viral infection, we cloned a paralog, termed BigPEN as it contained an additional RPT domain. All reported penaeidins from shrimp can be clustered into three subgroups, and each subgroup contained one or two penaeidins from *L. vannamei*. Specifically, PEN3 is located in subgroup 1; PEN2 and PEN4 are located in subgroup 2; and BigPEN is located in subgroup 3 (Figure 1). Since the structure of penaeidins is conserved within the subgroups, it is possible that each subgroup also has similar functions. In this model, the function of *L. vannamei* BigPEN, PEN2, PEN3, and PEN4 during WSSV infection could be representative, to some extent, of those penaeidins in other shrimps.

The name of penaeidins comes from the originality of their structure and the fact that they are only found in penaeid shrimps [48]. Penaeidins are composed of a conserved PEN domain, including a PRR and a CRR, which contains six cysteine residues that form three intramolecular disulfide bridges [49]. In this study, we identified BigPEN, which contained an RPT domain prior to the PEN domain. It was apparent that the RPT domain was not able to interact with the five tested viral proteins (Figure 4H and I) or the outer surface of WSSV virion (Supplementary Figure 2C). However, the actual function of the RPT domain is still unknown. In contrast, the PEN domain is conserved and has been demonstrated to be able to interact with one or more viral envelope proteins. Although PEN4 failed to interact with the five tested viral proteins, it may still interact with other viral proteins, as shown by its capability to bind to the outer surface of WSSV virions. This was further supported by rPEN4, which like the other three penaeidins, conferred hemocytes protection against WSSV entry.

The most probable scenario is that the antiviral activity of penaeidins is due to the direct interaction between the WSSV and penaeidins, which would inhibit viral entry into target cells. Our current studies have provided several lines of evidence strongly supporting this notion. First, all four penaeidins were able to interact with the outer surface of the WSSV virion, and all, except PEN4, were shown to bind one or more of the five tested viral proteins. Second, the purified penaeidin proteins inhibited WSSV entry into hemocytes and significantly reduced the infection rate of hemocytes by WSSV virions. Finally, PEN2 and BigPEN directly interacted with VP24 and VP28 of WSSV, respectively, and interfered with the binding of these viral proteins to host factors. The exact antiviral mechanism of penaeidins against WSSV could be their ability to disrupt the processes of viral infection. In this study, the identified penaeidin-binding viral proteins were VP24, VP26, VP28, and VP16 – most of which play significant roles in WSSV infection. VP24 is a chitin-binding protein and deemed to be a key factor involved in WSSV infection [50]. Importantly, VP24 has recently been identified as the viral receptor-binding protein, which has been shown to interact with the cellular receptor of MjpIgR from *M. japonicus* shrimp to mediate WSSV entry into host cells [14]. In this study, we also demonstrated that VP24 mediated WSSV penetration into non-permissive cells (HEK293T) via LvpIgR, indicating that LvpIgR from *L. vannamei* shrimp is a bona fide cellular receptor for WSSV internalization. VP26 was identified as an integral linker protein and was shown to bind to host actin to help transport virions into host cells [51]. VP28 is located on the outer surface of WSSV and was shown to be involved in viral attachment to and penetration of shrimp cells [52]. In addition, VP28 has been shown to interact with Rab7 of *P. monodon* (PmRab7), which has been shown to contribute to effective infection [13]. The interaction between VP28 and PmRab7 has been implicated in the WSSV infection process, most likely the step involving viral escape from the endosome [18]. We also observed that VP28 interacted with LvRab7, which has 100% sequence identity to PmRab7, indicating that the function of LvRab7 was similar to that of PmRab7. We provided evidence that PEN2 interacted with VP24 and interfered with VP24 binding to the viral receptor LvpIgR, thus inhibiting WSSV entry into cells. Additionally, BigPEN interfered with VP28 binding to LvRab7, which probably inhibited the trafficking of endosomes.

In addition, interactions between envelope proteins are common in enveloped viruses, and they might form complexes that have specific roles in host-viral interactions or the infectivity of viruses [3]. A similar situation has also been observed in the enveloped virus of WSSV. VP24, VP26, and VP28 share sequence homology and can form a complex termed an “infectosome” that has been regarded to be crucial to the infectivity of WSSV [53,54]. It is important to note that we are still unclear whether the interaction of penaeidins with viral proteins will interfere with the formation of an “infectosome” or other complexes involving host and viral proteins. Nevertheless, based on our results and previous observations, it is reasonable to conclude that the binding of penaeidins to viral envelope proteins attenuates WSSV infectivity and inhibits WSSV internalization.

A common innate defense mechanism in invertebrates, including shrimps, is that immune signalling pathways, such as Toll, IMD, and JAK/STAT pathways, regulate the production of specific sets of effectors for antiviral defense [44,47]. Identification of which pathway is responsible for the transcriptional expression of penaeidins in shrimps will help us better understand their mediated immune response to WSSV infection. Our results suggested that both Dorsal and Relish (NF-κB), the downstream transcription factors of Toll and IMD pathways, respectively, could be involved in the regulation of the four penaeidins after WSSV infection *in vivo*. This observation was further confirmed via an EMSA that showed that Dorsal was able to interact with the canonical κB motif within the promoter region of *BigPEN in vitro*. It is important to note that one or both κB motifs could also be responsive to Relish, as Relish strongly induced the expression of BigPEN *in vitro* as determined by dual reporter gene assays (Figure 10C). Similar situations have also been reported. For example, a κB motif within the promoter of WSSV IE1 (*wsv069*) has been demonstrated to be dual-responsive – that is, regulated by either Dorsal or Relish [55]. In *Drosophila*, a single κB motif in the promoter of Metchnikowin (*Mtk*) has been shown to bind both DIF and Relish [56]. Additionally, other signalling pathways may also regulate the expression of some penaeidins, as shown by the presence of several regulatory factor-binding motifs, such as NF-κB, GATA, STAT, and AP-1, within the promoter regions of penaeidins [57]. Thus, we propose that the Toll and IMD signalling pathways, perhaps via crosstalk with other pathways, work together in a collaborative manner to regulate the expression of penaeidins in response to WSSV infection. Such a regulatory pattern might be able to provide a rapid and tailored immune response against a viral invasion.

**Figure 11.**
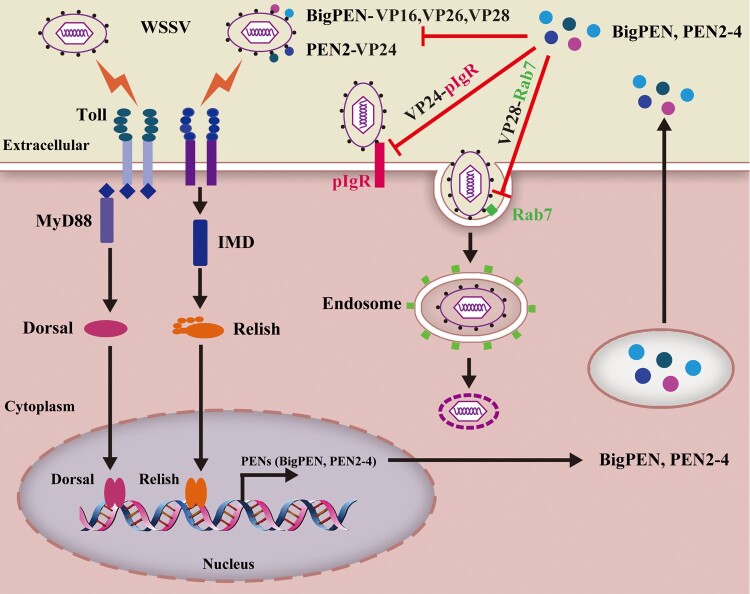
Model for penaeidins-mediated antiviral mechanisms against WSSV. Infection of host cells by WSSV results in activation of two NF-κB signalling pathways, which trigger the synthesis and secretion of penaeidins, including BigPEN, PEN2, PEN3, and PEN4. These secreted penaeidins restrict WSSV infection by interacting with the virion particles and antagonizing viral envelope proteins, which results in the blockade of multiple viral infection processes. In particular, PEN2 interferes with the receptor-binding protein VP24 to bind the host receptor LvpIgR, blocking viral entry into the target cells. In addition, BigPEN interferes with VP28-LvRab7 interaction, disrupting WSSV endocytosis.

In summary, for the first time we have identified penaeidins as a novel class of innate antiviral factors against WSSV. Based on our results, we proposed a model for the function of penaeidins in the innate antiviral response (Figure 11). Infection of host cells with WSSV results in the activation of the Toll and IMD (NF-κB related) signalling pathways that induce the production of penaeidins, including BigPEN, PEN2, PEN3, and PEN4. These secreted penaeidins restrict WSSV infection by antagonizing viral envelope proteins, which results in the blockade of multiple viral infection processes. Specifically, PEN2 interferes with the ability of the receptor-binding protein VP24 to bind to the host receptor LvpIgR, thereby blocking viral entry into the target cells. In addition, BigPEN interferes with VP28 binding to LvRab7 – a key regulator for viral endocytosis – which disrupts WSSV internalization. Furthermore, other penaeidins could abrogate infection by a similar mechanism. Therefore, this study provides insights into the development of antiviral agents that target viral infection processes.

## Supplementary Material

Supplemental Material

## Data Availability

All reagents and experimental data are available within the article or Supplementary Information or from corresponding author upon reasonable request.
