# Supplementary material for: Penaeidins restrict white spot syndrome virus infection by antagonizing the envelope proteins to block viral entry
Source: Emerg Microbes Infect. 2020 Feb 20;9(1):390–412. doi: 10.1080/22221751.2020.1729068 (PMC7048182; doi:10.1080/22221751.2020.1729068)
Supplement: Supplemental Material [file TEMI_A_1729068_SM6002.doc]

**Supplementary Table 1. Sequences of primers used in this research.**

| **Primers** | **Sequences (5′-3′)** |
| --- | --- |
| **RACE** | |
| UPM | CTAATACGACTCACTATAGGGCAAGCAGTGGTATCAACGCAGAGT |
| NUP | CTAATACGACTCACTATAGGGC |
| 3’RACE-F1 | TACCAGAGGACTTCAAGCAGACGAG |
| 3’RACE-F2 | AGCAGTGGACCTCAAGCA |
| 5’RACE-R1 | GGGTCTGTCTTCCTCCTGAAG |
| 5’RACE-R2 | CGAAGGTCGGAGTACAGGTC |
| **Quantitative PCR** | |
| BigPEN-RT-F | ACCACAGACCCCAAGTCCTA |
| BigPEN-RT-R | AGTTCCGGCAGATTTCGGTT |
| PEN2-RT-F | TGGTCTGCCAAGGCGAAG |
| PEN2-RT-R | AAGTGACAACAGCTTCCGAAC |
| PEN3-RT-F | TGTACAAGGGCGGTTACACG |
| PEN3-RT-R | CTTTCCCACGTGACAGCAAC |
| PEN4-RT-F | GGTCTGCCTGGTCTTCTTGG |
| PEN4-RT-R | CCCCGTATCTGAAGCAGCAA |
| EF-1α-RT-F | TATGCTCCTTTTGGACGTTTTGC |
| EF-1α-RT-R | CCTTTTCTGCGGCCTTGGTAG |
| **Absolute quantitative PCR** | |
| WSSV32678-F | TGTTTTCTGTATGTAATGCGTGTAGGT |
| WSSV32753-R | CCCACTCCATGGCCTTCA |
| TaqMan-probe-WSSV32706 | CAAGTACCCAGGCCCAGTGTCATACGTT |
| **Protein expression** | |
| BigPEN-FL-F | GAGGGGCCGCCTGGAGTGCTGCGTCCTC |
| BigPEN-FL-R | ACAGCAGGAGTTCCAGCGCTTGCAG |
| BigPEN-PEN-F | GGGAATTCTTTAAGCAGACCAGGCCTTCTTA |
| BigPEN-PEN-R | GGCTCGAGGGTTTGCTTGCCCGAGGAG |
| BigPEN-R-F | GGGAATTCCTCCAGGGACCGAGGAAGC |
| BigPEN-R-R | GGCTCGAGACAGCAGGAGTTCCAGCGCT |
| PEN2-F | CGGAATTCATGCGCCTCGTGGTCTGCC |
| PEN2-R | GGAAGCTTTTATCCTTTTACTAAGTGACAACAGC |
| PEN3-F | CGGAATTCATGCGCCTCGTGGTCTGCC |
| PEN3-R | CCCTCGAGTCAACCGGAATATCCCTTTCCC |
| PEN4-F | CGGGATCCATGCGCCTCGTGGTCTGCC |
| PEN4-R | CCCAAGCTTCTATCCTCTGTGACAACAATCCC |
| VP19-F | CGCGGATCCATGGCCACCACGACTAACAC |
| VP19-R | CCGCTCGAGTTAATCCCTGGTCCTGTTCTTAT |
| VP24-F | TACTCAGAATTCAACATAGAACTTAACAAGAAAT |
| VP24-R | TACTCACTCGAGGCCAGGAGAAAAACGCAT |
| VP26-F | TACTCAGAATTCACACGTGTTGGAAGAAGCGT |
| VP26-R | TACTCAGAATTCACACGTGTTGGAAGAAGCGT |
| VP28-F | TACTCAGAATTCATGGATCTTTCTTTCACTCT |
| VP28-R | TACTCACTCGAGTTACTCGGTCTCAGTGCCAG |
| VP16-F | GGATCCATGCCTGGAGCAATCACATTGAG |
| VP16-R | GAATTCTCAACTTCTACCATAAAGATATACTACAATG |
| Rab7-F | CGGGATCCATGGCATCTCGCAAGAAGATT |
| Rab7-R | CCCAAGCTTTTAGCAAGAGCATGCATCCTG |
| pIgR-F | CGGAATTCCAAGAGTACAGCGTAAATGAACCA |
| pIgR-R | CGGGATCCTCCGCTCAGGGTATCTTCAGA |
| **RNA interference** | |
| dsBigPEN-F | GGATCCTAATACGACTCACTATAGGGCCTATTGCTCGCCCTCA |
| dsBigPEN-R | GGATCCTAATACGACTCACTATAGGCGGCAGATTTCGGTTTCC |
| dsPEN2-F | GGATCCTAATACGACTCACTATAGGATGCGCCTCGTGGTCTGCC |
| dsPEN2-R | GGATCCTAATACGACTCACTATAGGTTATCCTTTTACTAAGTGACAACAGC |
| dsPEN3-F | GGATCCTAATACGACTCACTATAGGATGCGCCTCGTGGTCTGCC |
| dsPEN3-R | GGATCCTAATACGACTCACTATAGGATGCGCCTCGTGGTCTGCC |
| dsPEN4-F | GGATCCTAATACGACTCACTATAGGTCAACCGGAATATCCCTTTCCC |
| dsPEN4-R | GGATCCTAATACGACTCACTATAGGATGCGCCTCGTGGTCTGCC |
| dsDorsal-F | GGATCCTAATACGACTCACTATAGGCTATCCTCTGTGACAACAATCCC |
| dsDorsal-R | GGATCCTAATACGACTCACTATAGGATCTTTGACCTCATAGAAACGGAC |
| dsRelish-F | GGATCCTAATACGACTCACTATAGGCTGTTGACCCACCTTACCGAC |
| dsRelish-R | GGATCCTAATACGACTCACTATAGGAGAGGTGACAGAGGTGGGAT |
| dsGFP-F | GGATCCTAATACGACTCACTATAGGATGGTGAGCAAGGGCGAGGAG |
| dsGFP-R | GGATCCTAATACGACTCACTATAGGTTACTTGTACAGCTCGTCCATGCC |
| **Dual-luciferase** | |
| BigPEN-F | GGGGTACCCACATACACACACATATATATATGTGTCTGTC |
| BigPEN-R | CCGCTCGAG GTCTGTCCTCTTCGTGCTGATCAAG |
| BigPEN-F-M1 | GGGGTACCCGGAGAAAACATTATCAGACACAAACATATAT |
| BigPEN-F-M2 | GGGGTACCTAGTGTTTTTCGCCGGAGAAAACATTATC |
| BigPEN-R-M | CCGCTCGAG TCTGTCCTCTTCGTGCTGATCAAG |
| PEN2-F | GGGGTACCACTAGTTCCTTATTTTTATTTTATCGAT |
| PEN2-R | GGAGATCTCGCAGGAGGGAACCCGG |
| PEN3-F | GGGGTACCGACTACTGGAAATGTTTACGGTCCT |
| PEN3-R | GGAGATCTGGCGGACGCAGGAGGG |
| PEN4-F | GGGTACCACATGCAGATACAGATACATATATTCATATT |
| PEN4-R | GGAAGATCTGCGGACGCAGGAGGCAAC |
| **Genome walking** | |
| AP1 | GTAATACGACTCACTATAGGGC |
| AP2 | ACTATAGGGCACGCGTGGT |
| BigPEN -GW1 | GAAGGGTAAGCAGGTAGGAC |
| BigPEN -GW2 | GAAGGCCAAGACGAAAAGA |
| **Overexpression of LvpIgR in nonpermissive cells** | |
| pIgR-pcDNA3-F | CGGAATTCATGTGCAAGTGTTCCTTTGTGC |
| pIgR-pcDNA3-R | GGCTCGAGCTACTCCTTTTTCTCATCTTCCCG |
| Genomic DNA F | CCAACAAGTGTCTCCTCCAAAT |
| Genomic DNA R | Genomic DNA R AATCTCCTCAGGGATGTCAAAGT |
| **EMSA^a^** | |
| Bio-probe1 | TCTGTGTGTGTGTCCTTTAGTGTTTTTCGCCGGAGAAAACATTATCAGAC |
| Mut-bio-probe1 | TCTGTGTGTGTGTCCTTTACGGAGAAAACATTATCAGA |

**^a^ κB motif is underlined.**

**Supplementary figures and legends**

**Supplementary figure 1.** The interaction between rBigPEN and VP28 from WSSV infected hemocytes. The hemocytes were collected from 15 shrimp at 24 hours post WSSV infection. Then, the hemocytes were treated by RIPA lysis buffer 30 minutes at 4 °C, and the supernatant was collected by centrifugation at 5,000 × *g* for 10 minutes at 4 °C. The supernatant of 500 ul was co-incubated with Ni-NTA resin and 100 μg purified rBigPEN-FL or rTrx (as a control) 2 hours at 4 °C. The Ni-NTA resin was washed six times with PBS and subjected to western blotting using the VP28 antibody. The result showed that rBigPEN-FL could interact with the VP28 from the hemocyte lysates at 24 hours post WSSV infection.

**Supplementary figure 2. BigPEN-R did not interact with the surface of WSSV virion and block WSSV infection.** (A) BigPEN-R did not block WSSV infection. rBigPEN-R (the recombinant RPT domain of BigPEN) was incubated with the FITC-labeled WSSV (green) and then added into the hemocytes. Cells were fixed, permeabilized, and incubated with primary antibodies directed against actin protein (red), followed by incubation with the appropriate secondary antibodies. Cells were subsequently stained with DAPI (blue) and then observed under a fluorescent microscope. BSA protein was used as a control. Scale bar, 25 μm. (B) Statistical analysis of the WSSV infection-blocking rates of rBigPEN-R corresponding to (A). (C) rBigPEN-R did not interact with the surface of WSSV virion. The purified rBigPEN-R was first labeled with colloidal gold and then incubated with purified WSSV virions. After being stained with phosphotungstic acid, the viral suspension was adsorbed onto carbon-coated nickel grids and observed via transmission electron microscopy (TEM). The rTrx and BSA proteins were used as controls. Arrows show the locations of BSA, rTrx, and rBigPEN-R, which were labeled with colloidal gold. Scale bar: 50 nm. All experiments were performed three times with similar results. (D) The gates were created based on the hemocytes, and R1 was representative of intact hemocytes. (E) The influence of rBigPEN-R on the infection rate of hemocytes by FITC-labeled WSSV via flow cytometry. BSA and an rTrx protein were used as controls. Cells were examined by forward scatter (FSC, x-axis), and the infection rate of hemocytes was indicated by intracellular green fluorescence (y-axis). The scatter plots represent one of the three flow cytometric detections. (F) Statistical analysis of the infection rate of hemocytes corresponding to (E). All the data were analyzed statistically by Student’s *t* test (NS, not significant).

**Supplementary figure 3. PEN2 did not interact with LvpIgR, while BigPEN did not interact with PmRab7 or LvRab7.** (A-C) PEN2 did not interact with LvpIgR. (A) Recombinant expression and purification of the GST-tagged extracellular domain of LvpIgR (LvpIgR-SC). Lane 1, uninduced *E. coli* transformed with LvpIgR; lane 2, induced *E. coli* transformed with LvpIgR-SC; lane 3, supernatant of ultrasonic lysed *E. coli* expressing LvpIgR-SC; lane 4, purified GST-tagged LvpIgR-SC. (B–C) MBP-pulldown assay to detect the interaction between rPEN2 with LvpIgR-SC. PEN2 did not bind LvpIgR-SC, which was demonstrated by (B) Coomassie blue staining and (C) western blot analysis. (D–E) BigPEN did not interact with PmRab7 or LvRab7. His-tag pulldown assays to detect the interaction between BigPEN-FL (the full-length of BigPEN) with PmRab7 or LvRab7. BigPEN-FL did not bind with PmRab7 or LvRab7, as shown by (D) Coomassie blue staining and (E) western blot analysis.

**Supplementary figure 1**

**
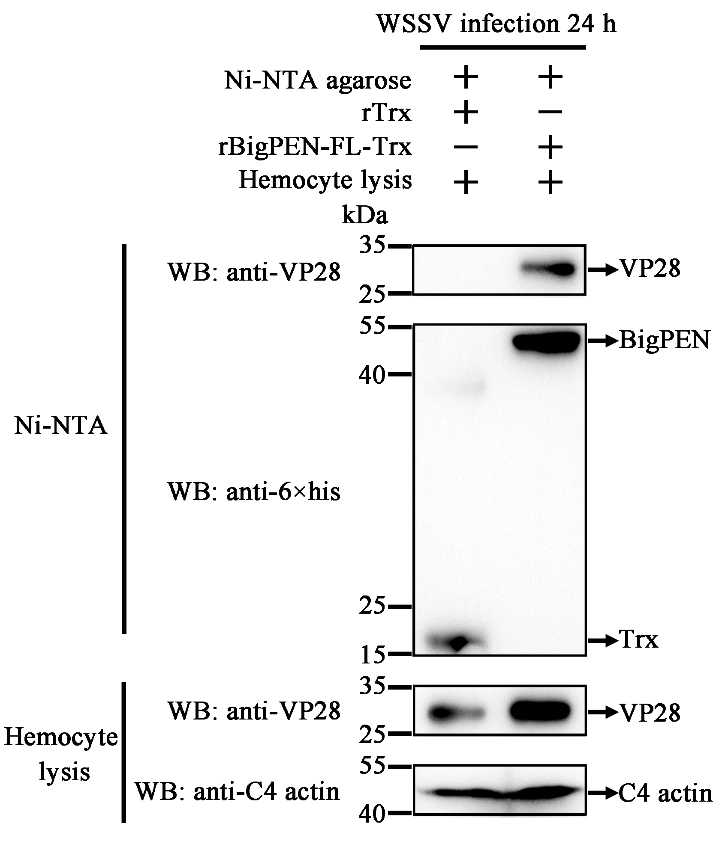
**

**Supplementary figure 2**

**
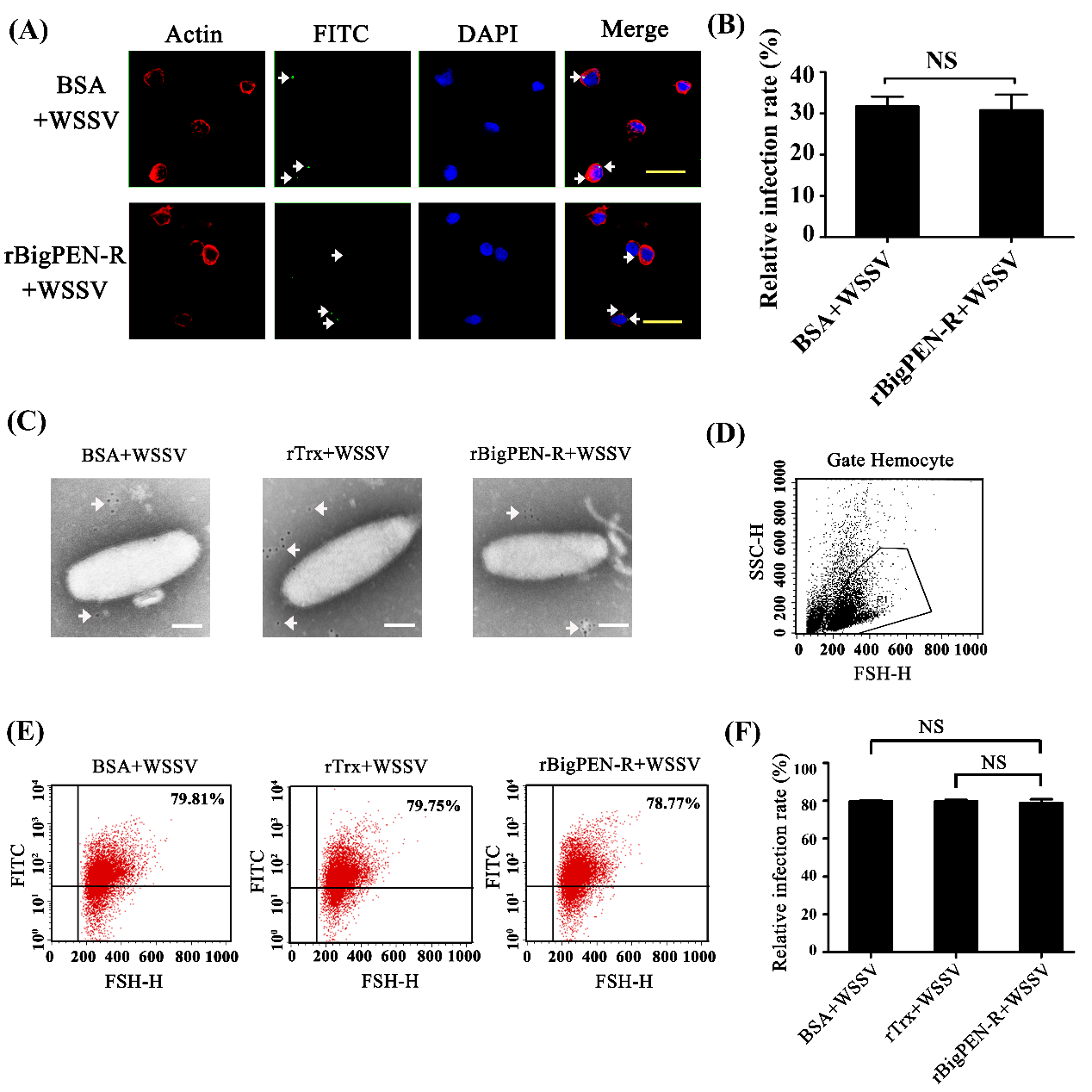
**

**Supplementary figure 3**

**
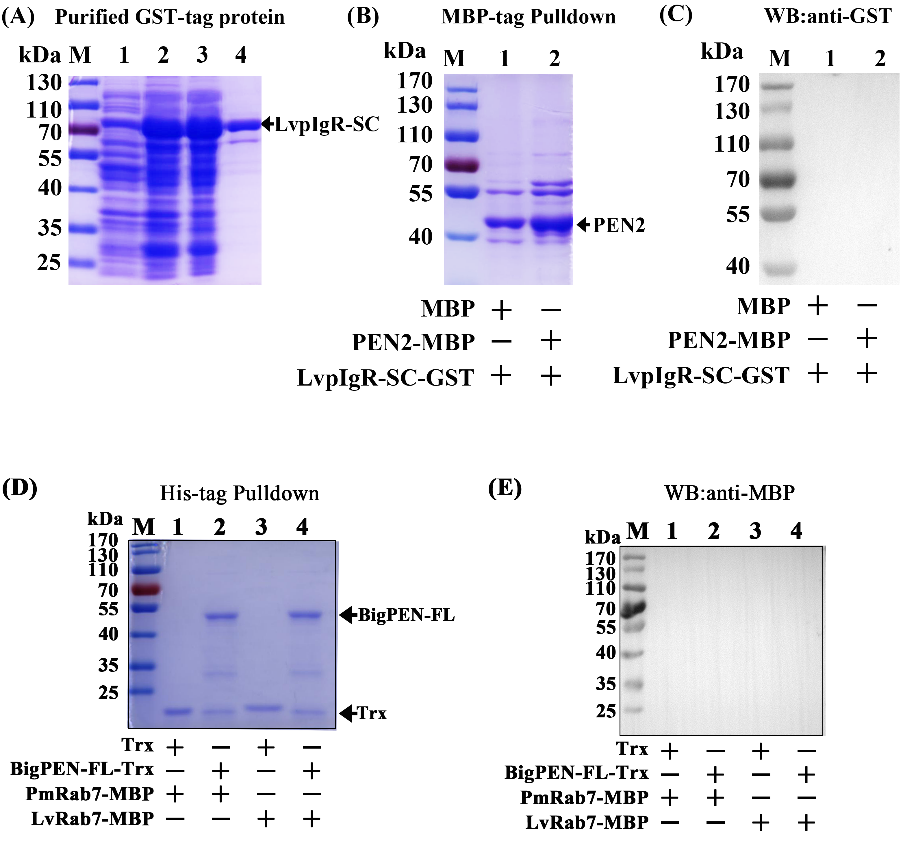
**

**Supplementary data 1. Partial promoter sequences and putative NF-κB binding sites of *BigPEN* and *PEN2*-4.** The putative NF-κB binding sites in the promoters are shadowed.

Partial promoter of BigPEN is obtained by Genome Walking method. The putative NF-κB binding sites in its promoter were shadowed, and the transcription start site (**G/A**) and the translation initiation site (**ATG**) were showed.

> Partial promoter region of BigPEN

TCTGTGTGTGTGTCCTTTA**GTGTTTTTCGC**CGGAGAAAACATTATCAGACACAAACATATATACACACATTAAACACATACATACATGCACACATCAAATATAATAAAACACACATCAAATACCCACATTCATAGACACATAAAACAAACACATACATAGACCCATCGACCCATCAACACACAAATACATCTAAACACGCACACACACAATGTTTACGTAATTAAACGCCCATACTCTCCAGGTGAGAGATAACGAAGGATGCCTACATGTATATGG**GTGTTTTTTAC**ATGCGACCGGACATGGCTCCTTCGTCCCACGGCTCCCCGTCCATAGGGGGCAGATATAAGGCGCAAGGGCGAGACAGTGC**G**ACATTCGGCTGCCATCCTCCAGAAGGAGCAGTCTTGATCAGCACGAAGAGGACAGAC**ATG**AAGGGTCTTTTCGTCTTGG

> Partial promoter region of PEN2

GCTCAAATAAGCGACTACTGGAAATGTTACGGTCCTGGCCCCGGGGTCGTTGCCTGTCGGCGGTCCTGCATATACATATACATACATACGACGCCTGAAGGTGCTTTCACAACCGCGTGGCTTCTCCATAAAAGGCATGGCACCACGGCCTCCGGTGCC**A**CTCGGCGCTTGGCTCTCCCTCGAGCCTCACCTGCAGAGACCGACGCTCCGAGCCCGGGTTCCCTCCTGCGTCCGCC**ATG**CGCCTCG

> Partial promoter region of PEN3

GACTACTGGAAATGTTTACGGTCCTGGCCCCGGGGTCGTTGCCTGTCGGCGGTCCTGCATATACATATACATATATACGACGCCTGAAGGTGCTTTCACAACCGCGTGGCTTCACCATAAAAGGCATGGCACCACGGCCTCCGGTGCC**A**CTCGGCGCTTGGCTCTCCCTCGAGCCTCACCTGCAGAGACCGACGCTCCGAGCCCGGGTTCCCTCCTGCGTCCGCC**ATG**CGCCTCG

> Partial promoter region of PEN4

ACATGCAGATACAGATACATATATTCATATTTATATAATAAGTATGTATTTATCTACCCATGCCTTTATATTTACAATAATAGATGCCTATATGTATGCGAGTCAGAATAGAGGGCAACCATAACGGAAACACACAACGCTCATCGGGTCATTGCAATCTGTTTGAAAACTTCCCTAAAGACGATCGCTAACAGTGTGACTATTGTGTAGTTTTAGGTGATCTTCATTACGAGTGTTATTTATGTTT**AGAAAATAAG**GGGTTTTAGATATCAAAATTGATGATTAACAAGGATTCACTC**GAAAAAAT**TATTTCATTTCTACCCTCTGTTCATTTGTTTATTTGTCTCATAGTCTGTTTAGCTATTTATTTCTCAGCCTGTCGTTCTGCTTAGCTACGTCACTTTATCTATCTGTCTCTCTTATCTCTCTTATCCCCCTTCTCTTTCTTTCTCCTACCCAGTTT**CCCGTTTTCCCTT**CCCTCCTATGCATC**CTTTTTTCTCTCT**CCCTTATGCCCTTTCGCTCTCTATATCTCTTCCATCCTGTCCTTTCTCTCTCCCAAGTTGCTTGCACAACCGCGCGGCGTCTCCATAAAAGGCATGGCACCGAAGCGTCCGGTGCC**A**CTCGGCGCTTGGCTCTCCCTCGAGCCTCACCTGCAGAGACCGACGCTCCGAGCCCGGGTTGCCTCCTGCGTCCGCC**ATG**CGCCTCGTGG
